# Among rural older adults, an apparent physical-activity contrast in nonblank medication-field presence was markedly attenuated when chronic-disease management registration was incorporated into the analysis

**DOI:** 10.3389/fphar.2026.1965382

**Published:** 2026-10-15

**Authors:** Kun Xu, Yiming Zhang, Yongheng Zhao, Xuefeng Xi, Gaixia Hou, Zhuangzhuang Guo, Limeng Liu, Yajuan Wang, Dehui Zhang

**Affiliations:** 1 School of Wushu, Henan University, Kaifeng, China; 2 Henan University, Institute of Gerontology, Kaifeng, China; 3 School of Physical Education and Health Sciences, Mudanjiang Normal University, Mudanjiang, China; 4 Dongcheng Community Health Service Center, Wangkui, China

**Keywords:** data provenance, exposure misclassification, medication data quality, older adults, pharmacoepidemiology, physical activity, primary care, real-world data

## Abstract

**Background:**

Medication fields created for chronic-disease management may mislead when reused as medication-exposure measures in broader older-adult populations. Using routine exercise-frequency as routine-recorded physical activity (PA), we compared PA + records (daily, at least weekly, or occasional exercise) with PA− records (no exercise) and examined how nonblank medication-field presence changed after management-registration context was incorporated.

**Methods:**

This cross-sectional study used 2,270 examination records from a 2025 rural primary-care program in northeastern China; PA was available for 2,226 records. The outcome was any nonblank text in the main-medication field. Modified Poisson regression estimated prevalence ratios (PRs) with small-sample-adjusted cluster-robust inference across 20 sites. Sequential models incorporated demographics, site, and four hypertension/diabetes management-registration categories. Logistic regression estimated standardized proportions and absolute differences. Medication text underwent secondary audit, including 200 randomly sampled retained entries independently reviewed by two pharmacists.

**Results:**

Nonblank medication fields occurred in 1,114/1,157 (96.3%) records with hypertension and/or diabetes management registration versus 27/1,113 (2.4%) with neither registration. Field presence was low without registration and near-complete with hypertension-only or dual registration; the diabetes-only stratum was small (n = 37). Overall field presence was 56.4% in PA+ and 42.8% in PA− records (PR 1.317, 95% CI 1.076–1.612). After registration context was incorporated, the PR was 1.001 (95% CI 0.960–1.043); full adjustment yielded 1.002 (95% CI 0.959–1.046). Standardized proportions were 51.26% and 51.14%, a difference of 0.12 percentage points (95% CI −2.07 to 2.30). Among 1,368 retained entries, archived labels classified 75.9% as antihypertensive, 14.5% as glucose-lowering, 0.5% as other cardiovascular, 1.7% as other medications, and 7.4% as unresolved. Inter-reviewer agreement for broad therapeutic class was 99.5% (κ = 0.982), and parser–manual-reference concordance was 99.0%; these assessments did not verify medication exposure.

**Conclusion:**

Nonblank main-medication-field recording was strongly patterned by chronic-disease management registration, and the apparent overall PA-related contrast was markedly attenuated after this context was incorporated. Blank fields cannot establish medication nonuse, and nonblank or consistently parsed text cannot establish complete, current medication exposure.

## 1 Introduction

Reliable medication information is essential for pharmaceutical care, medication safety, and pharmacoepidemiologic research in older populations, in whom multimorbidity and treatment complexity are common (Barnett et al., 2012; Maher et al., 2014; Young et al., 2022). Routine primary-care records are an important source of real-world medication information, but a medication field is suitable for secondary analysis only when its intended population, reference period, recording workflow, and semantic content are understood (Weiskopf and Weng, 2013; Verheij et al., 2018; Thai and Winterstein, 2024). Nonblank text does not necessarily represent a complete or clinically accurate medication list, whereas a blank field cannot automatically be interpreted as medication nonuse (Haneuse and Daniels, 2016; Sutherland et al., 2018; Beukelman et al., 2023).

Routine health data are products of healthcare and administrative processes rather than neutral research measurements (Agniel et al., 2018). Whether a field is requested, entered, updated, or left blank may depend on disease-management pathways, healthcare contact, staff workflow, and the original function of the record (Gionfriddo et al., 2021). Field presence and missingness may therefore encode the data-generation process and generate apparently meaningful associations when variables are reused outside their intended context (Tan et al., 2023). Fitness for secondary use consequently requires distinction among field availability, structural conformance, semantic completeness and plausibility, and external clinical validity (Kahn et al., 2016).

Within China’s National Basic Public Health Service framework, annual health management for residents aged 65 years or older includes lifestyle, chronic-disease management, treatment, and medication-related information (Liu et al., 2025; Yang et al., 2026). The exercise-frequency item primarily captures intentional exercise and does not comprehensively represent farming, occupational, household, transportation-related, or other non-exercise activity (Bull et al., 2020; Sattler et al., 2020). The routine main-medication item is intended to document principal medications used during the preceding year among people receiving long-term treatment for chronic disease (National Health and Family Planning Commission, 2017). Treating this field as a complete inventory of current medication use in all older adults may misclassify medication exposure before analyses of drug utilization, safety, effectiveness, or medication–outcome associations are conducted (Beukelman et al., 2023; Thai and Winterstein, 2024). Five previous analyses from the same or a substantially overlapping 2025 Wangkui County platform addressed disease phenotypes and screening-derived outcomes but did not primarily evaluate the source medication field itself (Gao et al., 2026; Guo et al., 2026; Liu et al., 2026; Zhang et al., 2026; Zhao et al., 2026b).

We evaluated whether the routine main-medication field could support secondary research among older adults. The routine exercise-frequency item was treated as routine-recorded physical activity (PA): daily exercise, exercise at least once per week, and occasional exercise were classified as PA+, whereas no exercise was classified as PA−. The primary statistical comparison contrasted nonblank medication-field presence between PA+ and PA− records before and after incorporating hypertension and diabetes management-registration context; PA was not treated as a causal determinant of medication use. Because the medication field was designed for chronic-disease management, field presence was also described by registration status to interpret the PA contrast within its data-generating context. A secondary text audit assessed what could be extracted from nonblank entries and which limitations remained. Additional *post hoc* descriptive audits examined medication-class correspondence using the archived dictionary and registered records with blank fields.

## 2 Materials and methods

### 2.1 Study design, setting, and data source

We conducted a cross-sectional pharmacoepidemiologic data-usability study using deidentified routine health-examination records maintained by Dongcheng Community Health Service Center in Wangkui County, Suihua, Heilongjiang Province, northeastern China. The records were generated through the government-organized 2025 annual health-management program for community-dwelling adults aged 65 years or older under China’s National Basic Public Health Service framework (National Health and Family Planning Commission, 2017). Examinations were recorded between March 17 and 28 October 2025, across 20 original primary-care examination sites represented in the database.

Wangkui County is a predominantly agricultural rural setting. Its geographic and socioeconomic characteristics were treated as part of the context in which the routine records were generated rather than as environmental exposures. Dongcheng Community Health Service Center provided the research team with a deidentified analytic export under institutional authorization.

Models used a deidentified analysis dataset with examination-record row identifiers. These identifiers were used for record linkage and audit within the dataset and were not treated as stable person-level identifiers. The verifiable unit of analysis was the examination record rather than a confirmed unique individual. The source workbook and record-level medication text were handled as controlled data.

The study evaluated the ascertainment and secondary-use suitability of a routine medication field. It was not designed to estimate a causal effect of physical activity on medication use or to evaluate medication effectiveness, safety, adherence, persistence, or clinical appropriateness. Operational definitions, coding rules, analytic roles, and interpretation boundaries are provided in Supplementary Table S1.

### 2.2 Record eligibility and analytic samples

All populated examination records for adults aged 65 years or older in the 2025 analytic export were eligible. The worksheet contained 2,270 populated examination records. Ten completely empty formatted rows at the end of the worksheet were ignored before analysis and were not treated as examination records or as excluded observations.

All 2,270 populated records were retained for descriptive analyses and classification of main-medication-field presence. Forty-four records with blank exercise-frequency information were excluded from the primary physical-activity analyses, leaving 2,226 examination records. For the fully adjusted model (defined in Section 2.8), two body mass index values outside the prespecified plausibility range of 12–60 kg/m^2^ were set to missing, resulting in 2,224 complete examination records.

Because unique individuals could not be independently verified from the deidentified export, two pairs of otherwise identical examination records were retained in the primary analyses. A sensitivity analysis retained only the first examination record within each pair. Missing values were not statistically imputed. Variable-level availability, quality flags, and the characteristics of records with missing physical-activity information are reported in Supplementary Table S2.

No *a priori* sample-size calculation was performed because the study included the complete set of available populated examination records meeting the data-source criteria.

### 2.3 Routine-recorded physical activity and exercise-frequency classification

The primary comparison variable was the routine exercise-frequency item, referred to throughout as routine-recorded physical activity (PA). Daily exercise, exercise at least once per week, and occasional exercise were classified as PA+, whereas no exercise was classified as PA−. These labels refer only to the recorded exercise item and should not be interpreted as validated classifications of total physical activity. Blank values were treated as missing and excluded from the primary PA comparisons because the comparison variable could not be classified.

The primary classification included the 11 records coded as exercise at least once per week. To reproduce the physical-activity harmonization used in a previous analysis of the same source platform, a sensitivity analysis classified daily and occasional exercise as PA+, classified no exercise as PA−, and excluded the weekly-exercise category (Gao et al., 2026; Guo et al., 2026; Liu et al., 2026; Zhang et al., 2026; Zhao et al., 2026b). A second alternative specification compared daily exercise with no exercise and excluded all other exercise-frequency categories and blank values.

Because the source item assessed exercise frequency rather than total movement behavior, routine-recorded physical activity was treated as a pragmatic database-derived behavioral indicator (Bull et al., 2020; Sattler et al., 2020). It was not interpreted as an objectively measured or comprehensive assessment of total physical activity. In particular, the item did not adequately capture occupational, agricultural, household, transportation-related, or other non-exercise activity, a distinction of particular relevance in an agricultural rural population.

Per-session exercise duration was summarized descriptively as recorded. The available fields did not support reliable derivation of exact weekly exercise frequency, total weekly exercise minutes, exercise intensity, metabolic-equivalent dose, or attainment of physical-activity guidelines.

### 2.4 Main-medication-field ascertainment

The National Basic Public Health Service specification defines the routine main-medication item as documenting principal medication use during the preceding year among people receiving long-term treatment for chronic disease (National Health and Family Planning Commission, 2017; health examination form completion instructions, item 10). The analytic export did not contain a record-level indicator confirming the reference period represented by each entry, and local adherence to the intended 1-year reference period could not be independently verified. The field was therefore not assumed to constitute a comprehensive population-wide inventory of current medication exposure. The implications of exposure windows in routinely collected healthcare data have been described elsewhere (Thai and Winterstein, 2024).

The primary outcome was field presence, defined as any nonblank text in the main-medication field. A blank field was an observed absence of text, not missing outcome data or evidence of medication nonuse. A nonblank field did not establish current medication use, a complete or accurate medication list, correct dosing, adherence, or treatment appropriateness, effectiveness, or safety. Discrepancies between recorded lists and independently assessed medication use are well documented (Sutherland et al., 2018; Beukelman et al., 2023).

### 2.5 Chronic-disease management registration and covariates

Hypertension and diabetes management registration were derived from the routine person-category field. These variables represented participation in chronic-disease management rather than independently adjudicated clinical diagnoses. They were treated as observable indicators of the documentation context in which medication information may have been more likely to be requested, reviewed, or entered and were not interpreted as biological mediators.

Management registration was represented by four mutually exclusive categories: neither hypertension nor diabetes registration, hypertension only, diabetes only, or both. This allows each combination to have its own estimated association with field presence, without assuming that hypertension and diabetes registration contribute additively on the log-prevalence scale. The four categories reflect the structure of the recorded information. Separate additive hypertension and diabetes indicators were examined in sensitivity analysis.

Additional covariates were age, sex, body mass index, current smoking, recorded alcohol use, and examination site. Age and body mass index were modeled continuously. Sex was coded as female *versus* male. Current smoking was coded as current smoking *versus* no current smoking, and recorded alcohol use was coded as any recorded use *versus* none. An alternative definition based on weekly or more frequent alcohol use was evaluated in sensitivity analysis.

For the fully adjusted model (defined in Section 2.8), body mass index values outside 12–60 kg/m^2^ were set to missing. The original body mass index values were retained without this plausibility restriction in a sensitivity analysis.

### 2.6 Examination-site structure

Twenty original examination sites were represented in the database. For examination-site fixed-effect adjustment and graphical display only, original sites contributing fewer than 20 examination records were combined into one anonymized pooled small-site category. All 20 original examination sites remained separate for CR2 and CR1 cluster-robust covariance estimation and for leave-one-original-site-out analyses.

Site-related analyses were undertaken to assess geographic or operational dependence, contextual variation, and the influence of individual original sites on the estimated physical-activity contrast. Site-level findings were descriptive and were not interpreted as rankings of documentation quality or service performance.

In an additional *post hoc* descriptive audit, registered records with blank medication fields were summarized by registration subtype, each original examination site, and recorded characteristics (Supplementary Table S9). Site-specific blank proportions used all registered records at that site as the denominator; sites without registered records were not assigned a percentage. Wilson intervals describe binomial proportions and do not adjust for within-site dependence. No site rankings, site-wise hypothesis tests, or high-dimensional blank-field regression were performed.

### 2.7 Audit of medication text and medication-class correspondence

The secondary text audit included all 1,141 records with nonblank medication fields. We standardized characters and separators using saved rules and split the text into 1,388 entries. Removing 20 exact duplicate complete entry strings within examination records left 1,368 retained entries. No entries were removed as duplicates across records or on the basis of inferred medication or ingredient identity. Each retained entry was a segmented text unit, not a confirmed unique medication or evidence of actual use. Complete rules are provided in Supplementary Methods S1.

We used a rule-based computer program to separate and classify medication text. The same text and rules produce the same output; saved versions of the code and dictionaries make changes traceable. The program identified medication names, quantities and units, dose-text structure, and duration or frequency information. Duration categories distinguished an explicit time unit, a bare number without a time unit, administration timing or frequency in the duration field, and other uninterpretable text. These checks assessed recorded information rather than clinical dose accuracy.

Technical implementation was evaluated using 48 prespecified synthetic test cases and exact row-level quality-control comparisons.

An additional *post hoc* medication-class analysis used confirmed labels in the archived project dictionary: antihypertensive for hypertension registration and glucose-lowering for diabetes registration (Supplementary Methods S1.13 and Supplementary Table S8). These project-specific labels defined the two target classes. Provisional or unresolved mappings were treated as unknown for both classes. A confirmed target-class identification was retained despite other unresolved text; otherwise any unresolved mapping made that class indeterminate. No corresponding target class identified in resolved recorded text did not establish actual nonuse. Analyses used all registered records and their nonblank subsets, with separate joint states for dual registration. The analysis did not establish verified exposure or treatment appropriateness.

To characterize the therapeutic content represented in the field, archived project-dictionary classes were summarized at both retained-entry and nonblank-record levels. These summaries were descriptive and were not interpreted as medication-use prevalence or as an externally validated therapeutic taxonomy.

The original development-stage pharmacist consultation was conducted jointly and did not retain reviewer-specific row-level decisions. To address this limitation, we subsequently conducted a revision-specific manual concordance assessment using a prespecified simple random sample of 200 of the 1,368 retained medication entries (14.6%). Two pharmacists independently reviewed the original recorded text while masked to parser outputs and to each other’s initial classifications. Reviewer-specific initial decisions were retained before inter-reviewer agreement was quantified. Disagreements were resolved by consensus or adjudication while parser output remained masked, and the resulting manual reference was locked before comparison with the frozen parser.

The manual assessment distinguished direct text extraction from semantic normalization. Prespecified domains included drug-name extraction, standardized medication naming, recorded-name certainty, ingredient-normalization certainty, broad therapeutic-class assignment, route extraction and interpretability, frequency, dose representation and unit, quantity-unit interpretation, duration category, and adherence text. Reviewers were instructed not to infer information absent from the recorded text.

This procedure assessed reproducibility and concordance in interpretation of recorded medication text. It did not constitute validation of actual medication exposure, medication-list completeness, dose correctness, adherence, or treatment appropriateness because no linked prescription, dispensing, medication-container, or structured medication-reconciliation source was available.

The frozen parser used for the reported validation was not substantively modified after review of the random validation sample.

### 2.8 Statistical analysis

Continuous variables were summarized as mean and standard deviation, and categorical variables as number and percentage. Absolute standardized mean differences were used to describe differences in record composition between PA+ and PA− groups. No hypothesis tests were used for these descriptive between-group comparisons.

Observed proportions of main-medication-field presence were reported with Wilson 95% confidence intervals (Newcombe, 1998b). The crude PA + -minus-PA− absolute difference in field presence was estimated with a Newcombe hybrid-score 95% confidence interval (Newcombe, 1998a).

Because nonblank fields were common, modified Poisson regression with a log link and robust variance was used to estimate prevalence ratios directly (Barros and Hirakata, 2003; Zou, 2004). The sequential models described how the crude activity-related contrast changed as increasingly detailed aspects of the recorded data-generating context were incorporated. They were not interpreted as a causal adjustment, mediation, or confounding-control sequence. Model 0 was unadjusted. Model 1 included age, sex, and examination-site fixed effects. Model 2 additionally incorporated the mutually exclusive four-category hypertension–diabetes management-registration context. Model 3 additionally included cleaned body mass index, current smoking, and recorded alcohol use.

Primary prevalence-ratio inference used CR2 cluster-robust covariance with Satterthwaite small-sample degrees of freedom across the 20 original examination sites (Pustejovsky and Tipton, 2018). HC0, HC3, and site-cluster CR1 covariance estimators were evaluated as sensitivity analyses. Modified Poisson fitted values were retained as model diagnostics and were not interpreted as individual-level probabilities.

To express the comparison as adjusted group proportions and an absolute percentage-point difference, we fitted a logistic regression model containing the same Model 3 covariates and performed marginal standardization (Muller and MacLehose, 2014). Physical-activity status was set in turn to PA+ and PA− for every complete examination record, while all remaining covariates retained their observed values. Standardized probabilities, the PA + -minus-PA− absolute difference, and the marginal prevalence ratio were reported with CR2-based delta-method confidence intervals and estimand-specific Satterthwaite small-sample degrees of freedom. These model-standardized contrasts were interpreted descriptively and not as causal effects.

Sensitivity analyses evaluated the additive two-indicator hypertension and diabetes registration specification; the previously published physical-activity harmonization excluding the weekly-exercise category; daily exercise *versus* recorded absence of exercise; classification of missing physical activity as PA−; alternative alcohol-use and body mass index specifications; candidate duplicate-record handling; exclusion of sites contributing fewer than 20 records; alternative covariance estimators; and sequential exclusion of each original examination site. Algebraic equivalence between the four-category representation and separate registration indicators plus their product term was treated as a coding quality-control check rather than as a substantive interaction analysis.

Additional analyses were descriptive and *post hoc*. We refitted all four models and calculated stage-specific logistic marginally standardized proportions and differences in the same 2,224 complete records (Supplementary Table S10), as a common-sample consistency check rather than a replacement for the primary models. Both deterministic coding scenarios for missing PA, all PA− and all PA+, were evaluated and reported regardless of their results. These scenarios do not correct missingness or define strict effect bounds. Registration-specific observed differences are also reported in Supplementary Table S10.

Sensitivity analyses and registration-category–specific analyses were interpreted descriptively, with emphasis on effect estimates and 95% confidence intervals rather than statistical significance. All reported tests were two-sided. No multiplicity adjustment was applied because no family of confirmatory subgroup hypotheses was specified.

Analyses for this revision were reproduced using R version 4.6.0 (R Foundation for Statistical Computing, Vienna, Austria), the archived project functions and the unchanged primary statistical specifications. Version-controlled code, aggregate outputs, numerical quality-control checks, synthetic parser-test results and the actual R session record were archived. Record-level data and medication free text remained in a controlled local archive. Numerical results were checked against the preceding analytic outputs before the new descriptive summaries and reuse illustration were assembled.

For the revision-specific manual assessment, inter-reviewer agreement was summarized as exact agreement with Wilson 95% confidence intervals. Cohen’s kappa with 95% confidence intervals was additionally reported for prespecified categorical classifications where meaningful. Because kappa may be affected by highly imbalanced category prevalence, raw agreement and the corresponding classification distributions were interpreted jointly. Indeterminate responses were retained as valid classifications, and no observations were excluded on the basis of reviewer confidence. No *post hoc* agreement threshold or category merging was used to improve agreement statistics.

Following masked resolution of reviewer disagreements, the manual reference was locked before parser output was unblinded. Domain-specific parser–manual-reference concordance was then summarized as n/N, percentage, and 95% confidence interval. Sensitivity, specificity, positive predictive value, negative predictive value, and clinical accuracy were not estimated because the manual reference represented interpretation of the same recorded text rather than an independent clinical medication source.

A quantitative reuse illustration applied the hypothetical interpretation of nonblank field presence as a medication-exposure proxy to the existing common sample of 2,224 complete records. We contrasted the apparent prevalence difference and marginal prevalence ratio before and after incorporating registration context, using the same logistic standardization framework. This illustration describes sensitivity of a field-derived proxy to documentation context; it does not estimate verified medication-use differences or the magnitude of corrected exposure misclassification.

### 2.9 Relationship with previous publications

The same or a substantially overlapping 2025 Wangkui County health-examination platform supported five previous publications addressing disease phenotypes and screening-derived outcomes (Gao et al., 2026; Guo et al., 2026; Liu et al., 2026; Zhang et al., 2026; Zhao et al., 2026b). The present study is a secondary analysis of that platform and not an independent cohort. Its distinct primary outcome is main-medication-field presence, supplemented by an entry-level medication-text audit; routine-recorded physical activity is used only as a recorded behavioral comparison of documentation patterns.

Study-level overlap, differences in analytic samples, the role of medication information in each report, and paper-specific distinctions are summarized in Supplementary Table S7. None of the previous reports treated main-medication-field presence or entry-level medication-text structure as the primary research object. An ethics-information correction was subsequently published for one previous report; the correction did not alter that report’s scientific analyses or original article DOI (Zhao et al., 2026a).

### 2.10 Ethics, data governance, and reporting

The study was approved by the Henan University Biomedical Research Ethics Sub-Committee (approval no. HUSOM2025-929; 12 November 2025). The source examinations were conducted as routine public-health services. For the present study, the research team received an existing deidentified analytic dataset, and the secondary analysis involved no additional participant recruitment, participant contact, intervention, or biological-specimen collection. The ethics approval document does not state that informed consent was waived for the present secondary analysis and does not document the consent procedure used for the original routine health examinations. Accordingly, we do not characterize the present study as having an ethics-approved informed-consent waiver or make a claim regarding original written informed consent on the basis of that document alone. The ethics approval document and data-authorization materials are retained in the controlled study archive.

The deidentified dataset was provided by Dongcheng Community Health Service Center under institutional authorization and with permission from the relevant local health authority. Names, addresses, direct personal identifiers, and original examination-site labels were not included in the analytic export. Record-level health variables and medication free text were treated as controlled data because residual re-identification risk could not be excluded. The study was conducted in accordance with the Declaration of Helsinki.

Reporting followed the Strengthening the Reporting of Observational Studies in Epidemiology (STROBE) statement, the REporting of studies Conducted using Observational Routinely-collected health Data (RECORD) statement, and the RECORD extension for pharmacoepidemiology (RECORD-PE) (Elm et al., 2007; Benchimol et al., 2016; Langan et al., 2018).

## 3 Results

### 3.1 Record flow, missingness, and analytic samples

The analytic export contained 2,270 populated examination records from adults aged 65–98 years. Routine-recorded physical-activity information was available for 2,226 records, of which 1,374 were classified as PA+ and 852 as PA−; 44 records had blank exercise-frequency information. The main-medication field was nonblank in 1,141 records. One field-present record had blank physical-activity information, leaving 2,226 examination records and 1,140 field-present records for Models 0–2. After two body mass index values outside the prespecified plausibility range of 12–60 kg/m^2^ were set to missing, Model 3 included 2,224 examination records and 1,139 field-present records (Figure 1).

**FIGURE 1:**
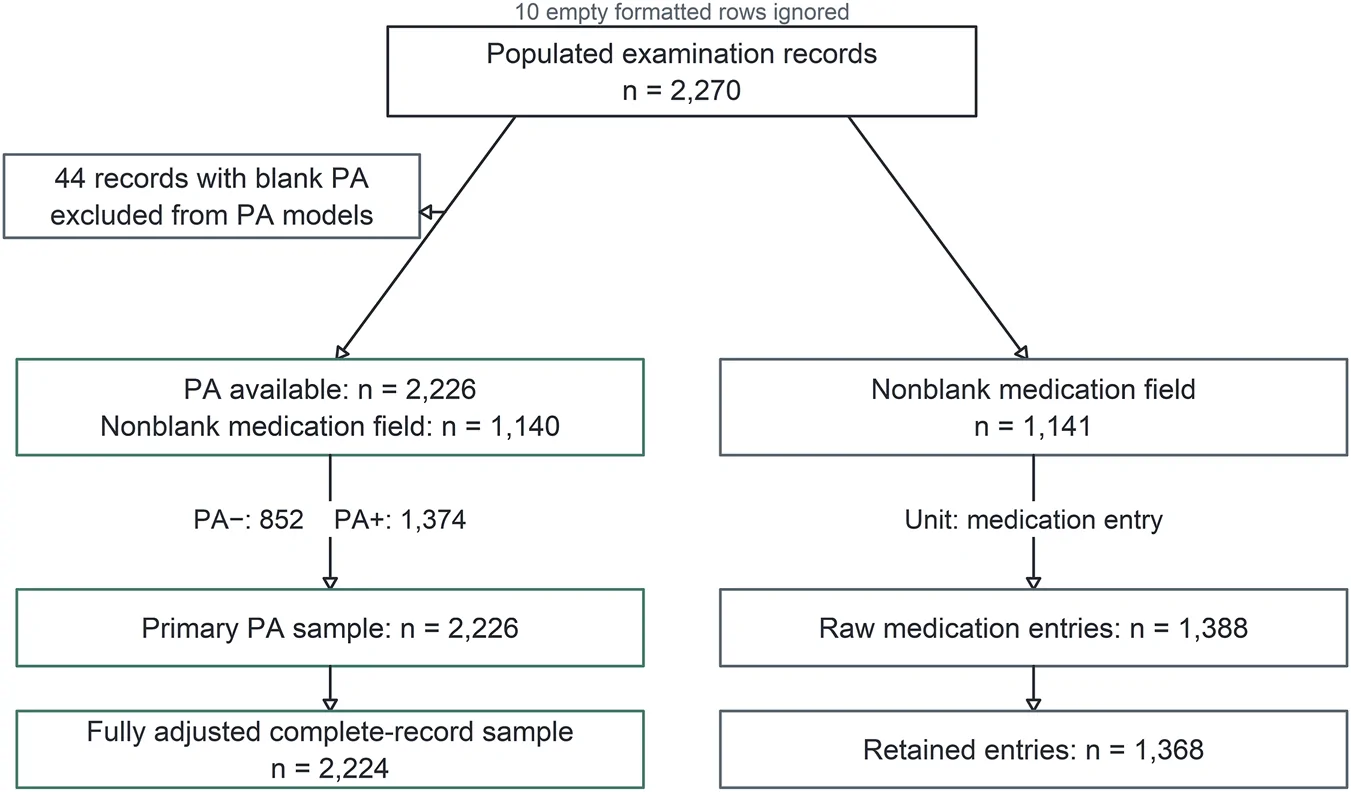
Study flow and final analytic units. The 2025 deidentified analytic export contained 2,270 populated examination records. Ten completely empty formatted rows at the end of the worksheet were ignored and were not treated as examination records. Routine-recorded physical-activity information was available for 2,226 records, including 852 PA− and 1,374 PA + records. The primary PA analysis sample included 2,226 records and 1,140 nonblank fields; the fully adjusted complete-record sample included 2,224 records and 1,139 nonblank fields. The medication-text audit included 1,141 records with a nonblank main-medication field and generated 1,388 raw medication entries. After removal of 20 exact duplicate complete entry strings within examination records, 1,368 medication entries were retained. Record-level analyses used the examination record as the unit of analysis, whereas the text audit used the parsed medication entry. No stable person-level identifier was available. PA denotes routine-recorded physical activity based on the routine exercise-frequency item; PA + indicates daily exercise, exercise at least once per week, or occasional exercise, and PA− indicates no exercise.

Among the 44 records with blank exercise-frequency information, one record (2.3%) had hypertension and/or diabetes management registration and one record (2.3%) had main-medication-field presence. The corresponding proportions among the 2,226 records with available physical-activity information were 51.9% and 51.2%, respectively. Characteristics of records with available and blank physical-activity information are reported in Supplementary Table S2.

### 3.2 Record characteristics and structure of the physical-activity measure

Among records with available physical-activity information, the mean age was 72.7 years and 51.2% were female. PA + records were younger than PA− records (71.6 vs. 74.5 years; absolute standardized mean difference, 0.483), had a higher mean body mass index (25.0 vs. 23.7 kg/m^2^; absolute standardized mean difference, 0.380), and more frequently had hypertension and/or diabetes management registration (57.5% vs. 43.0%; absolute standardized mean difference, 0.294). Current smoking and recorded alcohol use were also more frequent among PA + records (Table 1).

**TABLE 1:** Characteristics of examination records with available routine-recorded physical-activity information, overall and by physical-activity status.

Variable	Overall (N = 2,226)	PA− (n = 852)	PA+ (n = 1,374)	Absolute SMD
Age, years	72.7 (6.0)	74.5 (6.9)	71.6 (5.0)	0.483
Female sex, n (%)	1,139 (51.2)	432 (50.7)	707 (51.5)	0.015
BMI, kg/m^2^	24.5 (3.4)	23.7 (3.5)	25.0 (3.2)	0.380
Current smoking, n (%)	261 (11.7)	75 (8.8)	186 (13.5)	0.151
Any recorded alcohol use, n (%)	191 (8.6)	50 (5.9)	141 (10.3)	0.162
Hypertension management registration, n (%)	1,119 (50.3)	356 (41.8)	763 (55.5)	0.278
Diabetes management registration, n (%)	275 (12.4)	69 (8.1)	206 (15.0)	0.217
Hypertension and/or diabetes management registration, n (%)	1,156 (51.9)	366 (43.0)	790 (57.5)	0.294
Main-medication-field presence, n (%)	1,140 (51.2)	365 (42.8)	775 (56.4)	0.274

Values are mean (standard deviation) unless otherwise indicated. Body mass index summaries were based on available values; two examination records had missing body mass index after application of the prespecified plausibility rule. Absolute standardized mean differences compare PA+ with PA− examination records. No hypothesis tests were used for descriptive between-group comparisons. Hypertension and diabetes variables indicate routine chronic-disease management registration rather than independently adjudicated diagnoses. Main-medication-field presence indicates nonblank recorded text and does not establish clinically valid medication exposure, medication-list completeness, or clinical accuracy.

Abbreviations: BMI, body mass index; PA, routine-recorded physical activity based on the routine exercise-frequency item; SMD, standardized mean difference. PA + indicates daily exercise, exercise at least once per week, or occasional exercise; PA− indicates no exercise.

The original exercise-frequency field included 1,317 records indicating daily exercise, 46 indicating occasional exercise, 11 indicating exercise at least once per week, 852 indicating no exercise, and 44 with blank information. Positive per-session exercise duration was recorded for 1,366 of the 1,374 PA + records and was concentrated at 30 min (n = 222), 60 min (n = 801), and 120 min (n = 196) (Supplementary Figure S1).

### 3.3 Management-registration-specific medication-field presence

Across all 2,270 examination records, the main-medication field was nonblank in 96.3% of records with hypertension and/or diabetes management registration, compared with 2.4% of those with neither registration.

Among the 2,226 records with available PA, field presence was higher in PA + than in PA− records overall, with a crude difference of 13.6 percentage points (95% CI 9.3–17.8). The observed differences were much smaller within the two broad registration groups: neither registration and hypertension and/or diabetes registration (Supplementary Table S3).

The four mutually exclusive registration strata showed distinct recording patterns (Figure 2A; Supplementary Tables S4, 10). Among records with neither registration, field presence was 16/486 (3.3%) in PA− and 11/584 (1.9%) in PA + records. Corresponding values were 284/297 (95.6%) and 561/584 (96.1%) for hypertension-only registration, 6/10 (60.0%) and 25/27 (92.6%) for diabetes-only registration, and 59/59 (100.0%) and 178/179 (99.4%) for dual registration. The PA + minus PA− differences were −1.41, 0.44, 32.59, and −0.56 percentage points, respectively. The diabetes-only stratum contained only 37 records and was interpreted descriptively. No new interaction test was performed.

**FIGURE 2:**
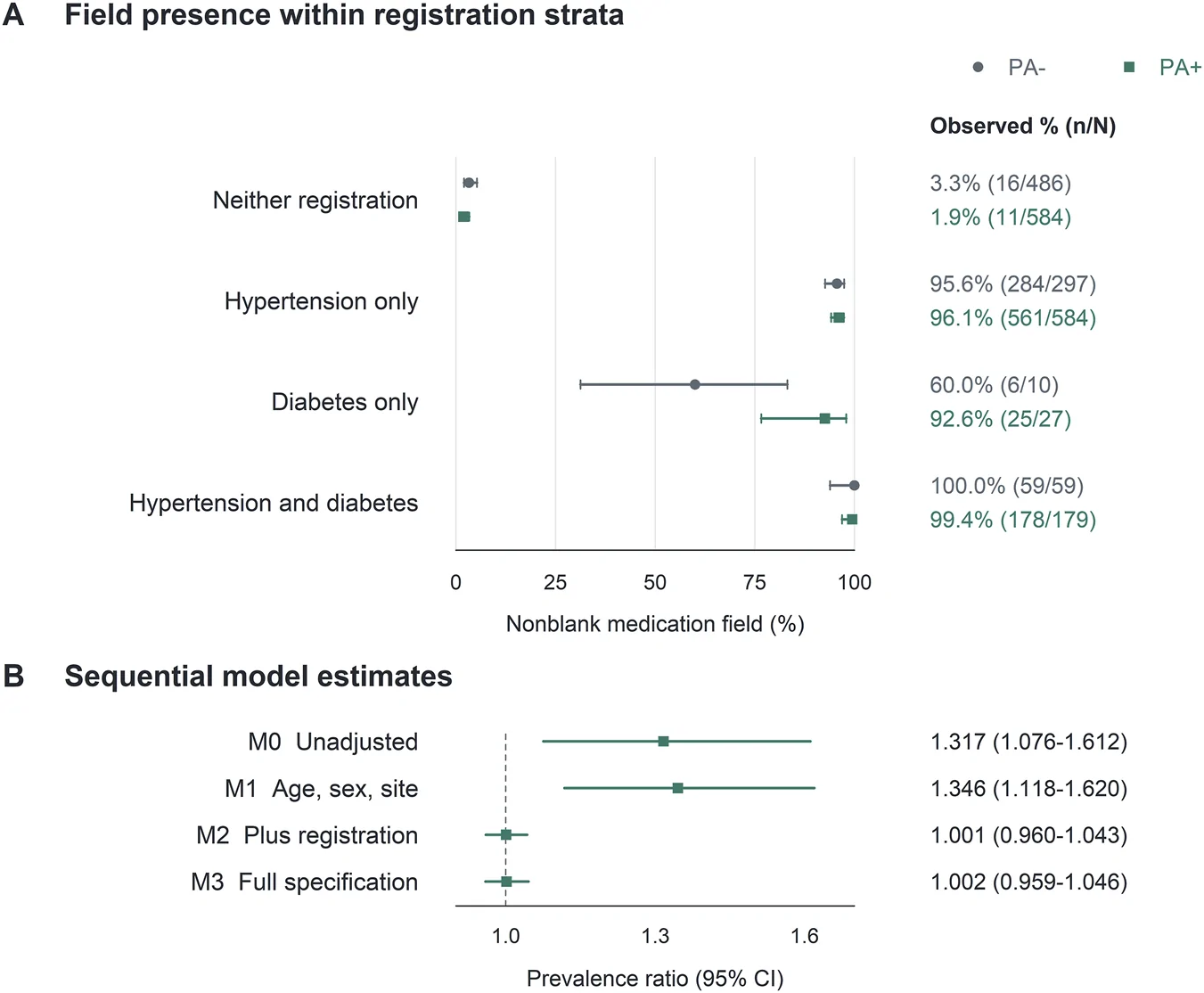
Medication-field presence within management-registration strata and sequential model estimates. Panel **(A)** reports observed PA− and PA + proportions with Wilson 95% confidence intervals and n/N labels in four mutually exclusive registration categories. These proportion intervals are descriptive and do not adjust for site clustering. The diabetes-only stratum is sparse. Panel **(B)** retains the original M0–M3 modified Poisson prevalence ratios with CR2/Satterthwaite confidence intervals across 20 original sites. Registration denotes recorded hypertension/diabetes management status, not adjudicated diagnosis. Nonblank text does not establish actual medication exposure.

### 3.4 Sequential regression models and marginally standardized estimates

The higher prevalence of nonblank fields in PA + records persisted after age, sex, and examination-site fixed effects were included, but the contrast was markedly attenuated when the four-category hypertension–diabetes management-registration variable was added. Adding cleaned BMI, current smoking, and recorded alcohol use made little difference to the estimate (Table 2, Panel A). Models 0–2 used the same 2,226 records, so the attenuation after incorporating registration did not reflect a change in the analysis sample.

**TABLE 2:** Sequential model estimates for routine-recorded physical activity and main-medication-field presence.

Model	Model specification	Examination records, N	Field-present records, n	PR	95% CI	P value
Panel A. sequential modified poisson regression models						
M0	Unadjusted	2,226	1,140	1.317	1.076–1.612	0.014
M1	Age, sex, and examination-site fixed effects	2,226	1,140	1.346	1.118–1.620	0.006
M2	M1 plus four-category hypertension–diabetes management-registration context	2,226	1,140	1.001	0.960–1.043	0.970
M3	M2 plus cleaned BMI, current smoking, and recorded alcohol use	2,224	1,139	1.002	0.959–1.046	0.932

Estimand	Examination records, N	Estimate	95% CI
Panel B. marginally standardized estimates from a logistic model with the model 3 covariates			
Standardized probability under PA− status	2,224	51.14%	49.77%–52.51%
Standardized probability under PA + status	2,224	51.26%	50.15%–52.37%
PA + minus PA− difference	2,224	0.12 percentage points	−2.07 to 2.30
Marginal prevalence ratio	2,224	1.002	0.960–1.046

Prevalence ratios compare PA+ with PA− examination records; PA− was the reference category. Model 0 was unadjusted. Model 1 included age, sex, and examination-site fixed effects. Model 2 additionally incorporated the four-category hypertension–diabetes management-registration context. Model 3 additionally included cleaned body mass index, current smoking, and recorded alcohol use. The sequential models described how the crude activity-related contrast changed as increasingly detailed aspects of the recorded data-generating context were incorporated; they were not interpreted as a causal adjustment, mediation, or confounding-control sequence. Primary prevalence-ratio inference used CR2 cluster-robust covariance with Satterthwaite small-sample degrees of freedom across the 20 original examination sites. Marginally standardized estimates were obtained from a logistic model containing the Model 3 covariates and used CR2-based delta-method confidence intervals with estimand-specific Satterthwaite small-sample degrees of freedom. Modified Poisson fitted values were retained only as model diagnostics and were not interpreted as individual-level probabilities. Sensitivity analyses and additional diagnostics are reported in Supplementary Table S5.

Abbreviations: BMI, body mass index; CI, confidence interval; CR1 and CR2, cluster-robust covariance estimators; M0–M3, sequential models; PA, routine-recorded physical activity based on the routine exercise-frequency item; PR, prevalence ratio. PA + indicates daily exercise, exercise at least once per week, or occasional exercise; PA− indicates no exercise.

Marginal standardization of the logistic model containing the Model 3 covariates yielded an overall PA + minus PA− difference close to zero (0.12 percentage points; 95% CI −2.07 to 2.30; Table 2, Panel B). This estimate describes field presence across the observed covariate distribution, not verified medication exposure.

### 3.5 Site-level descriptive analyses

The proportion of nonblank medication fields and record composition also varied across anonymized analysis-site categories. These displays were descriptive and were not interpreted as estimates of site effects or rankings of documentation quality or service performance (Supplementary Figure S3).

Among all 1,157 records with hypertension and/or diabetes registration, 43 had blank medication fields: 36/881 (4.1%) with hypertension only, 6/37 (16.2%) with diabetes only, and 1/239 (0.4%) with both. These records occurred across nine original sites. The two sites with nine blank records had 134 and 238 registered records, respectively (6.7% and 3.8%). Supplementary Table S9 provides site denominators and characteristics of the 43 blank and 1,114 nonblank registered records. These counts do not establish differences in care quality.

### 3.6 Sensitivity analyses and model diagnostics

The primary Model 3 prevalence ratio remained close to one across all alternative specifications. The estimate was 1.014 (95% confidence interval, 0.962–1.069) under the additive two-indicator hypertension and diabetes registration specification. Reapplication of the previously published physical-activity harmonization, which excluded the 11 records coded as exercise at least once per week, yielded a prevalence ratio of 0.998 (95% confidence interval, 0.954–1.045). The estimate was also 0.998 (95% confidence interval, 0.952–1.046) when daily exercise was compared with recorded absence of exercise and 1.004 (95% confidence interval, 0.962–1.048) when records with blank physical-activity information were classified as PA−.

Alternative alcohol-use and body mass index specifications, exclusion of candidate duplicate records, exclusion of sites contributing fewer than 20 records, and alternative covariance estimators yielded estimates close to the primary result. Leave-one-original-site-out estimates ranged from 0.993 to 1.012, and exclusion of any single original examination site did not materially alter the interpretation (Supplementary Table S5; Supplementary Figure S4).

All modified Poisson models converged. Maximum fitted values were 1.065 in Model 2 and 1.080 in Model 3; 148 and 194 fitted values, respectively, exceeded one. These fitted values were retained only as model diagnostics and were not interpreted as individual-level probabilities. The logistic Model 3 used for marginal standardization converged without non-finite coefficients, boundary estimates, or numerical separation warnings, and all model-based predicted probabilities were bounded between zero and one (Supplementary Table S5).

In the 2,224-record common sample, standardized PA + minus PA− differences across the four adjustment stages were 13.57, 14.54, −0.01, and 0.12 percentage points. The marked change after registration was added was therefore also seen on the absolute scale. When all 44 missing PA values were coded PA− or PA+, the full-model PRs were 1.004 (95% CI 0.962–1.048) and 0.999 (0.957–1.043), respectively; each scenario used 2,268 records after BMI exclusions (Supplementary Table S10).

### 3.7 Medication-text structure and medication-class correspondence

The medication-text audit retained 1,368 entries from 1,141 records with nonblank fields. Medication-name assignments remained provisional in 101 entries (7.4%). Duration information was frequently ambiguous: 537 entries (39.3%) contained an explicit time unit, 615 (45.0%) contained a bare number without a time unit, 212 (15.5%) contained timing or frequency information in the duration field, and four (0.3%) contained other or uninterpretable text. Detailed structural checks and technical test results are reported in Supplementary Table S6.

Across all retained entries, archived confirmed labels comprised antihypertensive (1,038/1,368; 75.9%), glucose-lowering (199/1,368; 14.5%), other cardiovascular (7/1,368; 0.5%), other medications (23/1,368; 1.7%). A further 101 entries (7.4%) remained provisional or unresolved. At record level, the corresponding class counts among 1,141 nonblank records were 954, 173, 7, and 22; 96 records contained at least one unresolved entry. Record-level categories overlap. The other cardiovascular group contained archived aspirin and simvastatin names, while other recorded names are listed without new therapeutic reclassification in Supplementary Table S11. These descriptive categories characterize the therapeutic content represented in the recorded field and should not be interpreted as population medication-use prevalence.

In the *post hoc* archived project-dictionary analysis, an antihypertensive target-class label was identified in 937/1,120 hypertension-registered records (83.7%) and a glucose-lowering target-class label in 167/276 diabetes-registered records (60.5%). Among nonblank records, the proportions were 937/1,083 (86.5%) and 167/269 (62.1%), respectively. Target-class status remained indeterminate in 77 hypertension-registered and 11 diabetes-registered records.

Among 1,114 registered records with nonblank fields, 14 (1.3%) had no corresponding target class identified in resolved recorded text and 69 (6.2%) were indeterminate. At least one class corresponding to at least one registered condition was identified in 1,031/1,157 registered records (89.1%); this did not require both classes in dual registration. Among 239 dual-registered records, both classes were identified in 73 (30.5%), the antihypertensive class in 168 (70.3%), and the glucose-lowering class in 137 (57.3%). Supplementary Table S8 reports the complete joint classification, including partial identification and uncertainty. These classifications did not verify actual medication exposure.

Technical reproducibility of the text rules does not establish medication-name accuracy or clinical validity; the available evidence and its limits are described in Supplementary Methods S1 and Supplementary Table S6.

In the revision-specific manual assessment, two pharmacists independently reviewed 200 randomly selected retained medication entries. Inter-reviewer exact agreement was 200/200 (100.0%; 95% CI approximately 98.1–100.0) for drug-name extraction, 181/200 (90.5%) for standardized medication naming, 192/200 (96.0%) for ingredient-normalization certainty, 199/200 (99.5%) for broad therapeutic-class assignment, 197/200 (98.5%) for route interpretability, 197/200 (98.5%) for quantity-unit interpretation, and 200/200 (100.0%) for duration classification. Broad therapeutic-class agreement was very high (κ approximately 0.98), and duration classification showed complete agreement (κ = 1.00). Ingredient-normalization certainty showed 96.0% raw agreement but a lower kappa because the underlying classification distribution was highly imbalanced. Full agreement results are reported in Supplementary Table S12.

After masked resolution of reviewer disagreements and locking of the pharmacist manual reference, concordance between the frozen parser and the manual reference was 200/200 (100.0%; 95% CI 98.1–100.0) for drug-name extraction, 198/200 (99.0%; 95% CI 96.4–99.7) for broad therapeutic-class assignment, 200/200 (100.0%; 95% CI 98.1–100.0) for quantity-unit interpretation, and 200/200 (100.0%; 95% CI 98.1–100.0) for duration classification. Standardized medication-name concordance was 155/200 (77.5%; 95% CI 71.2–82.7). These estimates quantify text-level concordance and do not establish actual medication exposure or clinical medication accuracy.

The 45 standardized-name discrepancies included formulation qualifiers, salt wording, combination-name formatting, and differences between broader archived dictionary labels and more specific manual names. Exact agreement was evaluated without post-review synonym substitution or removal of these qualifiers; disagreement therefore did not necessarily indicate a different medication identity. The two broad-class discrepancies and all discordant name pairs were retained in the comparison audit.

### 3.8 Quantitative illustration of naïve field reuse

If nonblank medication-field presence were naïvely treated as a population-wide medication-exposure proxy, the same 2,224-record sample would suggest apparent prevalences of 56.41% in PA+ and 42.84% in PA− records, a difference of 13.57 percentage points (95% CI 4.92–22.23) and a marginal PR of 1.317 (95% CI 1.075–1.613). After registration was incorporated in M2, the difference was −0.01 percentage points (95% CI −2.07 to 2.05) and the marginal PR was 1.000 (95% CI 0.960–1.041). In M3, these values were 0.12 percentage points (95% CI −2.07 to 2.30) and 1.002 (95% CI 0.960–1.046), respectively (Supplementary Table S13). These estimates illustrate the influence of documentation context on a field-derived proxy; they do not quantify actual medication-use differences or identify an unbiased corrected exposure estimate.

## 4 Discussion

Nonblank medication-field presence was strongly patterned by chronic-disease management registration. The overall PA-related contrast was markedly attenuated when this registration context was incorporated into the models. This pattern is consistent with the field’s intended chronic-disease use and illustrates how an apparent behavioral contrast can reflect the context in which information is recorded. It does not establish differences, or the absence of differences, in actual medication use. The text audit further showed that a nonblank field could still contain ambiguous medication information.

The additional correspondence analysis distinguished a nonblank field from identification of a condition-related target class in its recorded content. Neither establishes a complete current-medication list, actual exposure, or appropriate treatment. Differences across registered conditions must not be interpreted as undertreatment: the field was intended for principal medication information, and the archived project-specific mappings were not externally validated. Unresolved names and the conservative classification rules further limited interpretation.

The marked registration-related difference should not be interpreted simply as incomplete documentation of a medication list intended for all older adults. Under the National Basic Public Health Service specification, the source item was intended to document principal medication use during the preceding year among people receiving long-term treatment for chronic disease. Although local entry practices and record-level adherence to this reference period were not directly observed, the concentration of field presence among records linked to hypertension or diabetes management was compatible with a purpose-specific documentation process. A field may therefore be fit for its original operational purpose while remaining unsuitable for a different secondary-research purpose (Kahn et al., 2016; Verheij et al., 2018). Problems arise when such a field is treated as a comprehensive inventory of current medication exposure. A blank value may reflect inapplicability, lack of questioning or updating, unknown status, refusal, or omission, whereas nonblank text may represent historical rather than current use (Haneuse and Daniels, 2016; Verheij et al., 2018; Thai and Winterstein, 2024).

Hypertension and/or diabetes management registration was more common in PA + than in PA− records (Table 1), while field presence was near the upper boundary in both PA groups with hypertension-only or dual registration and low in both groups with neither registration. These patterns are consistent with the attenuation of the overall PA contrast after registration was incorporated. Near-ceiling field presence also limited variation in this binary outcome within the predominant registered groups. The overall standardized estimate should not be interpreted as exact equality or as excluding subgroup-specific differences or differences in actual medication use; the larger observed contrast in the sparse diabetes-only category remains descriptive. The sequential estimates do not identify a causal or mediation mechanism, and the near-null result alone does not establish inadequate statistical power.

PA was the main recorded comparison variable, but the exercise-frequency item did not measure total physical activity. It omitted agricultural, household, occupational, and other non-exercise activity relevant to this rural population (Bull et al., 2020; Sattler et al., 2020). Moreover, 1,317/2,226 records (59.2%) indicated daily exercise, and reported duration clustered at 30, 60, and 120 min. These patterns could reflect real routines, rounding or recall, questionnaire categories, or entry habits. We could not distinguish these explanations or verify the use of default entries. The daily-exercise comparison and other alternative PA definitions assess coding sensitivity, not the accuracy of the underlying activity measurement.

This distinction has broader relevance for studies combining behavioral variables with medication information derived from routine health records. Routine data are products of healthcare and administrative processes rather than neutral research measurements (Agniel et al., 2018; Verheij et al., 2018). Whether a medication field is requested, reviewed, updated, or left blank may depend on care pathways, management status, healthcare contact, staff responsibilities, and local workflow (Verheij et al., 2018; Gionfriddo et al., 2021). Informative field presence can consequently produce associations that reflect the documentation architecture of the database rather than the intended clinical construct (Haneuse and Daniels, 2016; Agniel et al., 2018; Kundu et al., 2024). In the present study, the four-category hypertension–diabetes registration variable captured one observable component of that architecture, but it did not represent the entire data-generating process. Other chronic diseases, medication-review encounters, staff practices, and local operational rules may also have influenced field completion. Because these processes were not directly observed, the findings support condition-patterned, context-dependent ascertainment but do not identify the precise local documentation rule.

Missingness in the physical-activity field was also strongly patterned by the recorded context. Among the 44 records with blank exercise-frequency information, only one had registered hypertension and/or diabetes and only one contained a nonblank main-medication field, compared with 51.9% and 51.2%, respectively, among records with available physical-activity information. This pattern is consistent with the possibility that missing behavioral information and medication-field completion arose partly from a shared documentation process (Haneuse and Daniels, 2016; Tan et al., 2023). Nevertheless, the small number of records with missing physical activity limits detailed interpretation, and the local reasons for missing entries were unavailable. Coding all records with missing PA as either PA− or PA + did not materially change the fully adjusted estimate. These deterministic scenarios assess coding sensitivity only; they do not identify or correct the underlying missingness mechanism.

The medication-text audit identified a further limitation of secondary use. Data-quality frameworks distinguish whether information is present, follows structural rules, is complete and plausible, and is independently verified (Weiskopf et al., 2013; Kahn et al., 2016; Lewis et al., 2023). Most entries met the implemented quantity–unit and dose-text rules, but this did not establish correct medication identification or clinically appropriate dosing. Ambiguous duration text further limited interpretation: a bare number does not specify a time period, and administration timing does not establish treatment duration (Supplementary Table S6).

The revision-specific manual assessment showed high inter-reviewer agreement and high parser–pharmacist concordance for direct text extraction and broad therapeutic-class assignment, whereas standardized medication naming showed greater variability than direct structural extraction. The frozen parser agreed with the manual reference on 77.5% of standardized names under exact normalized-text matching. This lower concordance reflected, in part, differences in the granularity and wording of archived dictionary labels and manually standardized names, and limits claims about reproducible semantic normalization. The lower inter-reviewer kappa for ingredient-normalization certainty occurred despite high raw agreement and was interpreted in the context of marked category imbalance.

Importantly, this assessment remained an internal validation of recorded-text interpretation rather than external clinical validation. Both pharmacists and the parser operated on the same source text, and no linked prescription, dispensing, medication-container, or structured medication-reconciliation source was available. High text-level concordance therefore cannot establish actual medication exposure, medication-list completeness, clinical-dose validity, or treatment appropriateness.

The common-sample illustration makes one practical consequence explicit: the same recorded field can produce a sizeable apparent medication-prevalence contrast when reused without its management-registration context. Holding the records constant excludes sample changes as the explanation for the change in that illustrative contrast. However, adjustment for registration does not recover unobserved true use, and registration does not fully determine the field’s intended population. The illustration therefore complements the descriptive strata rather than replacing exposure validation.

These limitations create several specific risks for pharmacoepidemiologic reuse. Coding blank fields as medication nonuse could produce differential exposure misclassification because field presence was strongly patterned by management registration (Beukelman et al., 2023; Thai and Winterstein, 2024). Restricting an analysis to records with nonblank medication fields could introduce selection bias by preferentially retaining records generated within hypertension or diabetes management pathways (Haneuse and Daniels, 2016; Kundu et al., 2024). The intended 1-year reference period may also be misaligned with outcomes measured at the examination visit or with analyses intended to represent current medication exposure (Thai and Winterstein, 2024). In addition, the number of parsed entries cannot be interpreted directly as the number of medications currently used: one retained entry does not establish monotherapy, and multiple retained entries do not establish polypharmacy. Adjustment for measured covariates cannot resolve these problems when the documentation process, exposure timing, and completeness of medication capture remain incompletely observed (Haneuse and Daniels, 2016; Kundu et al., 2024; Thai and Winterstein, 2024).

A tiered approach to secondary use is therefore more defensible. At the process level, field presence can support audits of documentation patterns, workflow dependence, and variation across management contexts. Within the population for which the field was originally intended, its content may support narrowly defined descriptive analyses when eligibility criteria, reference period, coding rules, and research question are aligned. In contrast, analyses of patient-level medication prevalence, polypharmacy, adherence, persistence, comparative effectiveness, safety, or medication–outcome associations require appropriate temporal alignment and validation against a suitable independent medication source (Public Policy Committee, International Society of Pharmacoepidemiology, 2016; Beukelman et al., 2023; Thai and Winterstein, 2024). Depending on the research question and local infrastructure, such sources may include prescriptions, dispensing records, structured medication reconciliation, medication-container review, or linked pharmacy data (Sutherland et al., 2018; Beukelman et al., 2023).

The findings also suggest practical improvements for routine primary-care information systems. A disease-management medication prompt should be distinguished from a comprehensive current-medication list. Medication identity, indication, formulation, strength, dose, unit, route, frequency, current status, start and stop dates, and information source should be stored in separate structured elements where feasible (Lima et al., 2026). Standardized terminology can further improve interoperability: RxNorm, a standardized clinical-drug terminology, supports normalized drug naming; the International Organization for Standardization Identification of Medicinal Products (ISO IDMP) standards support medicinal-product identification and exchange; and the Anatomical Therapeutic Chemical classification/Defined Daily Dose (ATC/DDD) system supports drug-utilization classification and aggregation (Nelson et al., 2011). These systems improve structure and comparability but do not, by themselves, establish that a recorded medication was actually taken during a defined exposure window (Thai and Winterstein, 2024). Explicit blank-value categories should also distinguish no long-term medication, not applicable, not asked, unknown, refused, and missing rather than representing all such states with an undifferentiated blank (Haneuse and Daniels, 2016; Verheij et al., 2018).

This study evaluates the medication field itself within the same or a substantially overlapping source platform used in previous publications; it is not an independent cohort replication. Its findings neither retrospectively validate nor invalidate medication-derived variables in those reports. Study overlap, sample differences, and the distinct research questions are described in Section 2.9 and Supplementary Table S7.

Several features strengthen the study. The analysis used the complete available deidentified export and explicitly distinguished examination-record-level analyses from medication-entry-level text auditing. The four-category hypertension–diabetes variable provided a saturated representation of the observed management-registration context and avoided imposing additivity between the two registrations on the log-prevalence scale. Primary prevalence-ratio inference used CR2 cluster-robust covariance with Satterthwaite small-sample adjustment across the 20 original examination sites, and both relative and marginal absolute estimates were reported. The interpretation was examined across alternative registration coding, physical-activity definitions, missing-value handling, body mass index and alcohol-use specifications, duplicate-record handling, variance estimators, small-site exclusion, and leave-one-original-site-out analyses. Modified Poisson fitted-value diagnostics were reported transparently, while probability-scale estimates were obtained independently through logistic marginal standardization. Finally, the medication-text workflow was version controlled and accompanied by predefined numerical checks, synthetic test cases, and exact row-level reproducibility comparisons.

The study also has important limitations. First, the cross-sectional design did not establish temporal ordering among physical-activity recording, management registration, medication-field completion, and actual medication use. Second, the records came from one community health service center in a single rural county, limiting transportability to other health-information systems and documentation workflows. Third, no stable person-level identifier was available, and examination records could not be independently confirmed to represent unique individuals. Fourth, the exercise-frequency item did not provide a comprehensive measure of physical activity. Fifth, hypertension and diabetes registration captured only part of the chronic-disease management and documentation context; other diagnoses and workflow factors were unavailable. Sixth, the registered-diabetes-only category contained few records, particularly among PA− examinations, and its descriptive contrast should not be generalized. Seventh, local medication-entry practices and adherence to the intended 1-year reference period were not directly observed. Eighth, inference relied on only 20 original examination-site clusters; CR2 small-sample adjustment and leave-one-site-out analyses reduced, but did not eliminate, uncertainty associated with a limited number of clusters (Pustejovsky and Tipton, 2018). Finally, although the revision-specific manual assessment provided internal evidence of inter-reviewer agreement and parser–pharmacist text-level concordance, it was not an external clinical validation study. The reviewers interpreted the same recorded medication text available to the parser, and no linked prescription, dispensing, medication-container, or structured medication-reconciliation source was available. The findings therefore do not establish actual medication exposure, medication-list completeness, clinical-dose validity, or parser transportability to other information systems.

The findings show why the purpose and meaning of a medication field need to be established before it is reused in research. Consistently parsed text is useful only when the field’s intended population, recording process, reference period, and clinical meaning match the proposed research question. These checks should precede analyses of drug utilization, safety, effectiveness, or medication–outcome associations.

## 5 Conclusion

Nonblank main-medication-field presence was concentrated among records linked to hypertension and/or diabetes management registration, consistent with the purpose-specific recording of this item. The crude PA-related contrast was markedly attenuated after the management-registration context was incorporated, and the fully standardized absolute difference was close to zero. These findings do not establish exact equality, absence of subgroup-specific differences, or absence of differences in actual medication use. For secondary research, a blank field cannot be assumed to indicate medication nonuse, and nonblank text cannot establish a complete current medication list. Field purpose, recording context, reference period, and medication-content uncertainty should therefore be assessed before this information is reused as medication exposure.

## Data Availability

The record-level health data and medication free text are not publicly available because residual re-identification risk cannot be excluded. Requests for access may be directed to the corresponding authors and will be considered subject to authorization by the data custodian and applicable institutional requirements.

## References

[B1] Agniel D. Kohane I. S. Weber G. M. (2018). Biases in electronic health record data due to processes within the healthcare system: retrospective observational study. BMJ 361, k1479. 10.1136/bmj.k1479 29712648 PMC5925441

[B2] Barnett K. Mercer S. W. Norbury M. Watt G. Wyke S. Guthrie B. (2012). Epidemiology of multimorbidity and implications for health care, research, and medical education: a cross-sectional study. Lancet 380, 37–43. 10.1016/S0140-6736(12)60240-2 22579043

[B3] Barros A. J. D. Hirakata V. N. (2003). Alternatives for logistic regression in cross-sectional studies: an empirical comparison of models that directly estimate the prevalence ratio. BMC Med. Res. Methodol. 3, 21. 10.1186/1471-2288-3-21 14567763 PMC521200

[B4] Benchimol E. I. Smeeth L. Guttmann A. Harron K. Hemkens L. G. Moher D. (2016). The REporting of studies conducted using observational Routinely-collected health data (RECORD) statement. Z Evid. Fortbild. Qual. Gesundhwes 115–116, 33–48. 10.1016/j.zefq.2016.07.010 27837958 PMC5330542

[B5] Beukelman T. Chen L. Annapureddy N. Oates J. Clowse M. E. B. Long M. (2023). Using pooled electronic health records data to conduct pharmacoepidemiology safety studies: challenges and lessons learned. Pharmacoepidemiol Drug Saf. 32, 969–977. 10.1002/pds.5627 37005701 PMC11246592

[B6] Bull F. C. Al-Ansari S. S. Biddle S. Borodulin K. Buman M. P. Cardon G. (2020). World Health Organization 2020 guidelines on physical activity and sedentary behaviour. Br. J. Sports Med. 54, 1451–1462. 10.1136/bjsports-2020-102955 33239350 PMC7719906

[B7] Elm E. von Altman D. G. Egger M. Pocock S. J. Gøtzsche P. C. Vandenbroucke J. P. (2007). The strengthening the reporting of observational Studies in Epidemiology (STROBE) statement: guidelines for reporting observational studies. Lancet 370, 1453–1457. 10.1016/S0140-6736(07)61602-X 18064739

[B8] Gao Y. Guo Z. Zhao Y. Xi X. Hou G. Liu L. (2026). Labour-type physical activity, metabolic dysregulation, and hypertension in rural older adults: rethinking work, exercise, and health in a cold-climate agricultural community. Front. Public Health 14, 1761980. 10.3389/fpubh.2026.1761980 41869631 PMC13003833

[B9] Gionfriddo M. R. Duboski V. Middernacht A. Kern M. S. Graham J. Wright E. A. (2021). A mixed methods evaluation of medication reconciliation in the primary care setting. PLoS One 16, e0260882. 10.1371/journal.pone.0260882 34855888 PMC8638945

[B10] Guo Z. Zhong Y. Zhao Y. Xi X. Hou G. Liu L. (2026). Routine-recorded physical activity and screening-defined cardio-renal-metabolic vulnerability amongst older adults in a cold-climate agricultural county in Northeast China. Front. Public Health 14, 1831345. 10.3389/fpubh.2026.1831345 42483053 PMC13386333

[B11] Haneuse S. Daniels M. (2016). A general framework for considering selection bias in EHR-based studies: what data are observed and why? EGEMS (Wash DC) 4, 1203. 10.13063/2327-9214.1203 27668265 PMC5013936

[B12] Kahn M. G. Callahan T. J. Barnard J. Bauck A. E. Brown J. Davidson B. N. (2016). A harmonized data quality assessment terminology and framework for the secondary use of electronic health record data. EGEMS (Wash DC) 4, 1244. 10.13063/2327-9214.1244 27713905 PMC5051581

[B13] Kundu R. Shi X. Morrison J. Barrett J. Mukherjee B. (2024). A framework for understanding selection bias in real-world healthcare data. J. R. Stat. Soc. Ser. A Stat. Soc. 187, 606–635. 10.1093/jrsssa/qnae039 39281782 PMC11393555

[B14] Langan S. M. Schmidt S. A. Wing K. Ehrenstein V. Nicholls S. G. Filion K. B. (2018). The reporting of studies conducted using observational routinely collected health data statement for pharmacoepidemiology (RECORD-PE). BMJ 363, k3532. 10.1136/bmj.k3532 30429167 PMC6234471

[B15] Lewis A. E. Weiskopf N. Abrams Z. B. Foraker R. Lai A. M. Payne P. R. O. (2023). Electronic health record data quality assessment and tools: a systematic review. J. Am. Med. Inf. Assoc. 30, 1730–1740. 10.1093/jamia/ocad120 37390812 PMC10531113

[B16] Lima V. Almeida R. Calvi I. Domingues D. Martins R. Bernardi F. (2026). From spreadsheets to FHIR and research databases: digitizing medication data in elderly care within the RM4Health project. Stud. Health Technol. Inf. 336, 1835–1839. 10.3233/SHTI260555 42175225

[B17] Liu L. Liu M. Yang L. Chen X. Liu Y. You L. (2025). A patient-reported questionnaire for evaluating utilization of the National Essential Public Health Service Program in China among older adults with hypertension and diabetes. Front. Public Health 13, 1459754. 10.3389/fpubh.2025.1459754 40135157 PMC11932853

[B18] Liu L. Zhao Y. Xuefeng X. Hou G. Liu Y. Wang Y. (2026). Diabetes-related exposure and screening-derived abnormality burden among older rural women in Northeast China: a secondary analysis with contextual labour-type physical activity assessment. Front. Public Health 14, 1841622. 10.3389/fpubh.2026.1841622 42459468 PMC13371314

[B19] Maher R. L. Hanlon J. Hajjar E. R. (2014). Clinical consequences of polypharmacy in elderly. Expert Opin. Drug Saf. 13, 57–65. 10.1517/14740338.2013.827660 24073682 PMC3864987

[B20] Muller C. J. MacLehose R. F. (2014). Estimating predicted probabilities from logistic regression: different methods correspond to different target populations. Int. J. Epidemiol. 43, 962–970. 10.1093/ije/dyu029 24603316 PMC4052139

[B21] National Health and Family Planning Commission of the People’s Republic of China (2017). “National basic public health service specifications,” in Health Examination Form and Completion Instructions; older-adult Health Management Service Specification. third edition. Beijing. Available online at: https://www.nhc.gov.cn/ewebeditor/uploadfile/2017/04/20170417104506514.pdf (Accessed August 11, 2026).

[B22] Nelson S. J. Zeng K. Kilbourne J. Powell T. Moore R. (2011). Normalized names for clinical drugs: rxnorm at 6 years. J. Am. Med. Inf. Assoc. 18, 441–448. 10.1136/amiajnl-2011-000116 21515544 PMC3128404

[B23] Newcombe R. G. (1998a). Interval estimation for the difference between independent proportions: comparison of eleven methods. Stat. Med. 17, 873–890. 10.1002/(sici)1097-0258(19980430)17:8<873::aid-sim779>3.0.co;2-i 9595617

[B24] Newcombe R. G. (1998b). Two-sided confidence intervals for the single proportion: comparison of seven methods. Stat. Med. 17, 857–872. 10.1002/(sici)1097-0258(19980430)17:8<857::aid-sim777>3.0.co;2-e 9595616

[B25] Public Policy Committee, International Society of Pharmacoepidemiology (2016). Guidelines for good pharmacoepidemiology practice (GPP). Pharmacoepidemiol Drug Saf. 25, 2–10. 10.1002/pds.3891 26537534

[B26] Pustejovsky J. E. Tipton E. (2018). Small-sample methods for cluster-robust variance estimation and hypothesis testing in fixed effects models. J. Bus. & Econ. Statistics 36, 672–683. 10.1080/07350015.2016.1247004

[B27] Sattler M. C. Jaunig J. Tösch C. Watson E. D. Mokkink L. B. Dietz P. (2020). Current evidence of measurement properties of physical activity questionnaires for older adults: an updated systematic review. Sports Med. 50, 1271–1315. 10.1007/s40279-020-01268-x 32125670 PMC7305082

[B28] Sutherland J. J. Morrison R. D. McNaughton C. D. Daly T. M. Milne S. B. Daniels J. S. (2018). Assessment of patient medication adherence, medical record accuracy, and medication blood concentrations for prescription and over-the-counter medications. JAMA Netw. Open 1, e184196. 10.1001/jamanetworkopen.2018.4196 30646345 PMC6324396

[B29] Tan A. L. M. Getzen E. J. Hutch M. R. Strasser Z. H. Gutiérrez-Sacristán A. Le T. T. (2023). Informative missingness: what can we learn from patterns in missing laboratory data in the electronic health record? J. Biomed. Inf. 139, 104306. 10.1016/j.jbi.2023.104306 36738870 PMC10849195

[B30] Thai T. N. Winterstein A. G. (2024). Core concepts in pharmacoepidemiology: measurement of medication exposure in routinely collected healthcare data for causal inference studies in pharmacoepidemiology. Pharmacoepidemiol Drug Saf. 33, e5683. 10.1002/pds.5683 37752827

[B31] Verheij R. A. Curcin V. Delaney B. C. McGilchrist M. M. (2018). Possible sources of bias in primary care electronic health record data use and reuse. J. Med. Internet Res. 20, e185. 10.2196/jmir.9134 29844010 PMC5997930

[B32] Weiskopf N. G. Weng C. (2013). Methods and dimensions of electronic health record data quality assessment: enabling reuse for clinical research. J. Am. Med. Inf. Assoc. 20, 144–151. 10.1136/amiajnl-2011-000681 22733976 PMC3555312

[B33] Weiskopf N. G. Hripcsak G. Swaminathan S. Weng C. (2013). Defining and measuring completeness of electronic health records for secondary use. J. Biomed. Inf. 46, 830–836. 10.1016/j.jbi.2013.06.010 23820016 PMC3810243

[B34] Yang L. Zhou Z. Lin L. Yan F. Yu L. Hu X. (2026). Multi-city elderly Health Examination Cohort Study (MEHECS) in China. Eur. J. Epidemiol. 41, 807–815. 10.1007/s10654-026-01409-y 42207416

[B35] Young R. A. Fulda K. G. Espinoza A. Gurses A. P. Hendrix Z. N. Kenny T. (2022). Ambulatory medication safety in primary care: a systematic review. J. Am. Board Fam. Med. 35, 610–628. 10.3122/jabfm.2022.03.210334 35641040 PMC9730343

[B36] Zhang K. Liu X. Ma C. Zhao Y. Xi X. Hou G. (2026). The “Futile Labour” Paradox: occupational physical activity fails to offset diabetes burden and is associated with microvascular stress signals in rural older adults. Front. Public Health 14, 1790766. 10.3389/fpubh.2026.1790766 41919304 PMC13033493

[B37] Zhao Y. Hou G. Gu Y. Guo Z. Zhang D. Xi X. (2026a). Correction: labour-type physical activity, alcohol use and hypertension in rural older adults in Northeast China. Front. Public Health 14, 1821356. 10.3389/fpubh.2026.1821356 41929900 PMC13041713

[B38] Zhao Y. Hou G. Gu Y. Guo Z. Zhang D. Xi X. (2026b). Labour-type physical activity, alcohol use and hypertension in rural older adults in Northeast China. Front. Public Health 14, 1748721. 10.3389/fpubh.2026.1748721 41822924 PMC12975895

[B39] Zou G. (2004). A modified poisson regression approach to prospective studies with binary data. Am. J. Epidemiol. 159, 702–706. 10.1093/aje/kwh090 15033648

