# Supplementary material for: Among rural older adults, an apparent physical-activity contrast in nonblank medication-field presence was markedly attenuated when chronic-disease management registration was incorporated into the analysis
Source: Front Pharmacol. 2026 Oct 15;17:1965382. doi: 10.3389/fphar.2026.1965382 (PMC13716648; doi:10.3389/fphar.2026.1965382)
Supplement: Supplementary file 1 [file Supplementaryfile1.docx]

Supplementary Methods S1. Version-controlled medication-text lineage, parsing, and quality-control specification

S1.1 Field provenance and reference period

The source was the routine main-medication field in the 2025 older-adult examination export. The National Basic Public Health Service specification defines this item for principal medication use during the preceding year among people receiving long-term treatment for chronic disease (National Health and Family Planning Commission, 2017; health examination form completion instructions, item 10, printed p. 14). Local record-level compliance with the intended one-year reference period could not be verified and was not assumed.

S1.2 Analysis units and field-presence outcome

The primary outcome was any nonblank text in the main-medication field. Record-level models used examination records; text auditing used retained medication entries. A parsed entry is a segmented text unit and must not be described as a unique medication or as evidence of actual use.

S1.3 Deidentification and row lineage

The controlled export retained record_id, raw medication text, its nonblank indicator, extraction version, and a SHA-256 digest of record_id plus text for linkage checks. Names, addresses, and real site labels were excluded.

S1.4 Entry segmentation

Chinese semicolons were normalized. Fields were split at semicolons, whitespace trimmed, and empty segments removed, yielding 1,388 entries from 1,141 nonblank records.

S1.5 Within-record exact-duplicate handling

Within each record, the first occurrence of each exact complete entry string was retained. Removing 20 duplicates left 1,368 entries; no cross-record or inferred ingredient-level deduplication was performed.

S1.6 Structural field extraction

Parser version 2 extracted labeled drug name, route, frequency, per-administration dose, duration/timing, and adherence after normalizing Chinese commas. Structural completeness required the expected labels in order; ambiguous clinical content was not repaired.

S1.7 Drug-name mapping and ambiguity

Raw names were joined to a versioned 161-row dictionary. Existing mappings and provisional or unresolved statuses were kept distinct, and ambiguous strings were not forced to an ingredient. Complete dictionary coverage is a technical property, not a medication-name accuracy estimate.

S1.8 Quantity and unit parsing

The parser used a versioned quantity–unit lexicon covering mass, solid-count, container, volume, and biologic units, including U and IU. A technical dose screen required a numeric value plus a recognized unit, excluded zero or negative values, and flagged solid or container counts above five. It was not a clinical-dose screen.

S1.9 Route, frequency, and adherence handling

Recorded route, frequency, and adherence strings were retained without clinical reinterpretation. Frequency tokens were not converted to duration. Lexical flags must not be interpreted as verified behavior.

S1.10 Duration and timing classification

Mutually exclusive duration-text categories were explicit time unit, bare number without unit, timing or frequency rather than duration, missing, and other or uninterpretable. The external timing lexicon included meal-related terms, PC, administration-count terms, clock-hour expressions, and clock times. Deterministic priority rules prevented timing tokens from being classified as duration.

S1.11 Pharmacist consultation and evidence boundary

The original development-stage pharmacist consultation was conducted jointly and did not retain reviewer-separated row-level decisions. This limitation applied to the development-stage review. A separate revision-specific manual concordance assessment was subsequently performed as described in Supplementary Methods S1.14 and Supplementary Table S12. No external clinical medication source was available.

S1.12 Reproducibility and quality-control locks

For the archived analysis version, executable reproduction locks required 2,270 records, 1,141 nonblank fields, 1,388 raw entries, 20 removed within-record duplicates, 1,368 retained entries, 1,357 recognized quantity–unit tokens, 1,323 technical dose-screen passes, and duration counts of 537/615/212/4 for explicit/bare/timing/other. All 48 synthetic cases and exact row-level comparison with the locked technical reference had to pass. These locks demonstrate technical reproducibility, not clinical validation.

S1.13 Archived project-dictionary target-class labels

Retained medication entries were linked by raw drug name to drug_dictionary_v2.csv, the archived 161-row project dictionary. Only mappings marked “Existing mapping confirmed” contributed identified target classes: the therapeutic_group labels “降压药” (antihypertensive) and “降糖药” (glucose-lowering) defined the hypertension and diabetes targets, respectively. No new ingredient-level normalization or combination-product decomposition was performed. These project-specific labels were not an externally validated therapeutic classification.

For each target separately, Identified required at least one confirmed mapping with that target label. No corresponding target class identified in resolved recorded text required no target label and all recorded medication-name mappings to be confirmed. Indeterminate required no confirmed target identification and at least one provisional or unresolved mapping; all such mappings were conservatively unknown for both targets. Field blank meant no recorded medication text. A confirmed target-class identification was retained even when another string in the same record remained unresolved.

HTN and DM statuses were generated separately for every record. For any registration, identification meant at least one target class corresponding to at least one registered condition, not necessarily both conditions in dual registration. Without such identification, uncertainty in any registered-condition target produced an indeterminate result; otherwise nonblank resolved text was classified as no corresponding target class identified. Blank fields remained separate. Denominators were all registered records and, separately, their nonblank subsets. Table S8C reports dual-registration joint states and overlapping marginal summaries.

The post hoc target-class analysis retained the archived mappings; the separate revision-specific manual assessment is reported in Supplementary Methods S1.14 and Table S12. Dynamic checks verified exhaustive states, registration and class margins, and denominators. A separate version snapshot verified current input, code and output counts; changed mappings require a new documented version. Code, input hashes, record-level statuses, joint counts and both QC reports are archived. The official field specification is documented in the National Health and Family Planning Commission (2017), National Basic Public Health Service Specifications, third edition, health examination form completion instructions (item 10, printed p. 14) and older-adult health management chapter.

**Supplementary Table S1. Operational definitions, coding rules, analytic roles, and interpretation boundaries**

| **Domain** | **Variable or rule** | **Operational definition** | **Analytic role or interpretation boundary** |
| --- | --- | --- | --- |
| Study design | Design | Cross-sectional pharmacoepidemiologic data-usability study using de-identified routine primary-care health-examination records. | Study design |
| Study setting | Data source | Records maintained by Dongcheng Community Health Service Center, Wangkui County, Heilongjiang Province, northeastern China, under the National Basic Public Health Service framework. | Rural primary-care data-generating context |
| Study period | Examination dates | March 17 to October 28, 2025. | Temporal setting of source records |
| Source population | Eligible records | All populated 2025 examination records for adults aged 65 years or older in the analytic export. | Source analytic population |
| Analysis unit | Examination record | The export contained no stable person-level identifier; one examination record per unique individual could not be independently confirmed. | Mandatory reporting boundary; records were not assumed to represent verified unique individuals |
| Database flow | Populated examination records | The worksheet contained 2,270 populated examination records. Ten completely empty formatted rows at the end of the worksheet were ignored and were not treated as excluded records. | Source-record flow |
| Primary analytic sample | Physical-activity-complete records | Records with nonmissing exercise-frequency information; N = 2,226. | Primary PA models and Table 1 |
| Model 3 analytic sample | Model 3 complete records | Records with complete PA and Model 3 covariates after two BMI values outside 12–60 kg/m² were set to missing; N = 2,224. | Model 3 relative and marginal estimates |
| Comparison variable | Primary recorded PA comparison variable | PA+ included daily exercise, exercise at least once per week, or occasional exercise. PA− included no exercise. Forty-four blank values were treated as missing and excluded from the primary PA models. | Primary recorded behavioral comparison; not interpreted as a causal exposure for medication use |
| Comparison boundary | Meaning of recorded physical activity | Routine-recorded intentional exercise rather than objectively measured total physical activity. The field did not comprehensively capture occupational, agricultural, household, transportation-related, or other non-exercise activity. | Not an exercise-dose, MET, total-activity, or guideline-attainment measure |
| Comparison-variable sensitivity | Alternative physical-activity definitions | The prior PA harmonization classified daily and occasional exercise as PA+, no exercise as PA−, and excluded exercise at least once per week; the second definition compared daily exercise with no exercise and excluded non-daily and blank values. | Alternative PA-definition sensitivity analyses |
| Comparison-variable missingness | Missing PA coding scenarios | The original sensitivity analysis coded missing PA as PA−. Additional post hoc scenarios coded all missing PA as PA− and, separately, PA+ (Table S10); neither corrects missingness or constitutes formal effect bounds. | Missing-data sensitivity analysis |
| Primary outcome | Main-medication-field presence | Presence of any nonblank text in the routine main-medication field. | Primary outcome |
| Outcome provenance | Intended field use | The source specification targeted principal medication use during the preceding year among people receiving long-term treatment for chronic disease (National Health and Family Planning Commission, 2017); record-level adherence to that reference period was not verified. | Purpose-specific field; not a population-wide current-medication inventory |
| Outcome boundary | Meaning of field presence | A blank field indicated absence of recorded text and was not interpreted as medication nonuse. A nonblank field did not establish actual use, medication-list completeness, medication-name accuracy, dose correctness, adherence, appropriateness, effectiveness, or safety. | Mandatory interpretation boundary |
| Data-generating context | Registered hypertension | Binary indicator derived from routine chronic-disease management registration. | Component of the four-category context and additive-coding sensitivity; not an adjudicated diagnosis |
| Data-generating context | Registered diabetes | Binary indicator derived from routine chronic-disease management registration. | Component of the four-category context and additive-coding sensitivity; not an adjudicated diagnosis |
| Data-generating context | Any registered hypertension and/or diabetes | Presence of registered hypertension, registered diabetes, or both. | Broad descriptive grouping only |
| Primary context variable | Four-category registration background | Neither registration; registered hypertension only; registered diabetes only; or both registrations. | Primary representation in Models 2 and 3; saturated management-context representation |
| Covariate | Age | Age in completed years, modeled continuously. | Models 1–3 |
| Covariate | Sex | Recorded sex, coded as female versus male. | Models 1–3 |
| Covariate | Body mass index | Weight in kilograms divided by height in meters squared. Values outside the defined range of 12–60 kg/m² were set to missing in primary Model 3. | Model 3 specification |
| Covariate sensitivity | Uncleaned body mass index | Original BMI values retained without the plausibility restriction. | Sensitivity analysis |
| Covariate | Current smoking | Current smoking versus no current smoking. | Model 3 specification |
| Covariate | Recorded alcohol use | Any recorded alcohol use versus none. | Model 3 specification |
| Covariate sensitivity | Weekly or frequent alcohol use | Alternative alcohol-use definition based on weekly or more frequent recorded consumption. | Sensitivity analysis |
| Examination site | Fixed-effect site variable | Sites contributing fewer than 20 records were combined into one anonymized analysis category. | Models 1–3 and graphical display |
| Examination site | Original-site clustering | All 20 original examination sites were retained separately for CR2, CR1, and leave-one-original-site-out analyses. | Primary CR2 inference and site-related sensitivity analyses |
| Duplicate-record handling | Candidate exact duplicates | Two pairs of otherwise identical examination records were retained because unique individuals could not be verified. | Primary analysis |
| Duplicate-record sensitivity | First record retained | Only the first record within each candidate duplicate pair was retained. | Sensitivity analysis |
| Missing-data handling | Primary approach | Complete-record analysis for variables required by each model; no statistical imputation was performed. | Primary analyses |
| Descriptive comparison | Absolute standardized mean difference | Absolute SMD comparing PA+ and PA− records. | Descriptive comparison; no baseline hypothesis testing |
| Observed proportions | Wilson confidence interval | Wilson 95% confidence intervals for observed field-presence proportions. | Descriptive interval estimation |
| Absolute crude contrast | Newcombe confidence interval | Newcombe hybrid-score interval for the PA+-minus-PA− difference in proportions. | Crude absolute effect |
| Relative effect | Modified Poisson regression | Log-link Poisson generalized linear model with robust sandwich variance; prevalence ratios were reported. | Primary relative effect measure |
| Sequential model | Model 0 | Unadjusted association between routine-recorded PA and main-medication-field presence. | Crude model |
| Sequential model | Model 1 | Model 0 plus age, sex, and examination-site fixed effects. | Model including demographic and site terms |
| Sequential model | Model 2 | Model 1 plus the mutually exclusive four-category hypertension–diabetes management-registration background. | Model representing the observed management context |
| Sequential model | Model 3 | Model 2 plus cleaned BMI, current smoking, and recorded alcohol use. | Most extensive model specification |
| Model interpretation | Sequential modeling | Models 0–3 described changes in the PA contrast as recorded covariates were added; they were not a causal adjustment or mediation sequence. | Mandatory interpretation boundary |
| Primary variance | CR2 with Satterthwaite degrees of freedom | Bias-reduced cluster-robust covariance across all 20 original examination sites with coefficient-specific small-sample degrees of freedom. | Primary prevalence-ratio inference |
| Variance sensitivity | HC0 and HC3 | Heteroskedasticity-consistent covariance estimators without site clustering. | Sensitivity analyses |
| Variance sensitivity | Site-cluster CR1 | Cluster-robust covariance across the 20 original examination sites with a t distribution using 19 degrees of freedom. | Sensitivity analysis |
| Marginal effect scale | Logistic marginal standardization | Logistic Model 3 with PA set in turn to PA+ and PA− for every complete record while other covariates retained observed values. | Standardized probabilities, absolute difference, and marginal prevalence ratio |
| Marginal uncertainty | CR2 delta method | CR2 covariance with estimand-specific Satterthwaite small-sample degrees of freedom. | Primary interval estimation for marginal estimands |
| Model-specification QC | Equivalent saturated coding | Four-category coding was checked against separate registration indicators plus their product term. | Algebraic coding check; not a substantive interaction analysis |
| Site influence | Leave-one-original-site-out analysis | Model 3 was refitted after sequential exclusion of each of the 20 original examination sites. | Influence and robustness assessment |
| Medication-text unit | Medication entry | Individual entry parsed from a nonblank main-medication-field string. | Entry-level analysis unit |
| Medication-text processing | Entry deduplication | Only exact duplicate complete medication-entry strings within the same examination record were removed. | Programmatic preprocessing rule |
| Medication-text audit | Dictionary assignment | Rule-based assignment of retained medication strings using a version-controlled dictionary. | Technical coverage; not validated medication-name mapping |
| Medication-text audit | Provisional medication-name flag | Indicator that a medication-name assignment remained provisional or uncertain under the rule-based dictionary. | Programmatic uncertainty indicator |
| Medication-text audit | Quantity–unit token | Presence of a numeric quantity accompanied by a recognizable unit token. | Structural conformance; not clinical dose accuracy |
| Medication-text audit | Technical dose-syntax screen | Deterministic screen defined in parser version 2 for numeric dose and recognized quantity–unit syntax. | Technical syntax assessment; not dose plausibility or appropriateness |
| Medication-text audit | Explicit duration with time unit | Duration text containing an interpretable time unit. | Programmatic duration category |
| Medication-text audit | Bare number without time unit | Numeric duration entry without an interpretable time unit. | Semantically incomplete duration category |
| Medication-text audit | Administration timing or frequency | Administration timing or frequency information entered in the duration field. | Non-duration semantic category |
| Medication-text boundary | Pharmacist consultation and clinical-validation boundary | The original joint development-stage consultation was followed by a separate revision-specific manual assessment of 200 retained entries (S1.14; Table S12). No external clinical medication source was available. | Internal recorded-text agreement and concordance; not clinical exposure validation |
| Statistical software | R environment | R 4.6.0 for statistical analyses and additional descriptive audits; archived project functions. | Reproducibility information |
| Reproducibility | Archived workflow | Version-controlled code, deidentified outputs, numerical QC locks, parser test results, and R session information were archived. | Computational reproducibility |

**Notes:** Definitions of the annual health-management fields and their intended use followed the National Basic Public Health Service specification (National Health and Family Planning Commission, 2017). The verifiable unit was the examination record. PA was used as a recorded behavioral comparison of secondary field reuse and not as a causal determinant of medication use. Detailed parsing rules appear in Supplementary Methods S1; missingness is reported in Table S2, observed proportions in Tables S3–S4, sensitivity analyses in Table S5, the medication-text audit in Table S6, and previous-publication overlap in Table S7.

**Abbreviations:** BMI, body mass index; CR1 and CR2, cluster-robust covariance estimators; HC0 and HC3, heteroskedasticity-consistent covariance estimators; M0–M3, sequential models; PR, prevalence ratio; QC, quality control; SMD, standardized mean difference. PA denotes routine-recorded physical activity based on the routine exercise-frequency item; PA+ indicates daily exercise, exercise at least once per week, or occasional exercise, and PA− indicates no exercise.

**Supplementary Table S2. Data availability, final quality flags, and comparison of examination records with available versus missing routine-recorded physical activity**

**Panel A. Data availability and final quality flags**

| **Variable or data-quality item** | **Examination records assessed, N** | **Unavailable or flagged, n (%)** | **Primary analytic handling** |
| --- | --- | --- | --- |
| Routine-recorded physical activity | 2,270 | 44 (1.9) | Records with blank exercise-frequency information were excluded from primary PA analyses. The original PA− coding sensitivity was retained; additional all-PA− and all-PA+ scenarios are in S10. |
| Age | 2,270 | 0 (0.0) | Complete; no imputation |
| Sex | 2,270 | 0 (0.0) | Complete; no imputation |
| Cleaned BMI | 2,270 | 2 (0.1) | Values outside 12–60 kg/m² were set to missing; original values were retained in sensitivity analysis. |
| Current smoking | 2,270 | 0 (0.0) | Complete; no imputation |
| Recorded alcohol use | 2,270 | 0 (0.0) | Complete; no imputation |
| Registered hypertension | 2,270 | 0 (0.0) | Complete; no imputation |
| Registered diabetes | 2,270 | 0 (0.0) | Complete; no imputation |
| Main-medication-field-presence indicator | 2,270 | 0 (0.0) | Every record was classified as containing nonblank text or having a blank field; blank fields were not treated as missing outcome data. |
| Candidate exact duplicate examination records | 2,270 | 4 (0.2) | Two pairs were retained in the primary analysis because unique individuals could not be verified; only the first record within each pair was retained in a sensitivity analysis. |

**Panel B. Characteristics of examination records with available versus missing routine-recorded physical activity**

| **Characteristic** | **PA available (n = 2,226)** | **PA missing (n = 44)** | **Absolute SMD** |
| --- | --- | --- | --- |
| Age, years | 72.7 (6.0) | 72.0 (5.9) | 0.116 |
| Female sex, n (%) | 1,139 (51.2) | 24 (54.5) | 0.068 |
| BMI, kg/m² | 24.5 (3.4) | 24.1 (2.8) | 0.130 |
| Current smoking, n (%) | 261 (11.7) | 9 (20.5) | 0.239 |
| Any recorded alcohol use, n (%) | 191 (8.6) | 2 (4.5) | 0.163 |
| Registered hypertension and/or diabetes, n (%) | 1,156 (51.9) | 1 (2.3) | 1.347 |
| Nonblank main-medication field, n (%) | 1,140 (51.2) | 1 (2.3) | 1.327 |

**Notes:** Values in Panel B are mean (standard deviation) or n (%). BMI summaries were calculated among records with available cleaned BMI values; the two BMI values set to missing occurred among records with available PA information. Absolute SMDs were used only to describe differences between records with available and missing PA information; no hypothesis tests were performed. The large SMDs for chronic-disease registration and medication-field presence indicate that missing PA information was strongly patterned by the recorded data-generating context. They should not be interpreted as causal associations. A blank main-medication field was classified as absence of recorded text and not as missing outcome data or evidence of medication nonuse. No statistical imputation was performed.

**Abbreviations:** BMI, body mass index; PA, routine-recorded physical activity based on the routine exercise-frequency item; SMD, standardized mean difference. PA+ indicates daily exercise, exercise at least once per week, or occasional exercise; PA− indicates no exercise.

**Supplementary Table S3. Observed main-medication-field presence overall and within broad chronic-disease management-registration contexts**

**Overall and broad management-registration context**

| **Registration context** | **PA−, n/N** | **Field presence, % (95% CI)** | **PA+, n/N** | **Field presence, % (95% CI)** | **PA+ minus PA−, percentage points (95% CI)** |
| --- | --- | --- | --- | --- | --- |
| Overall | 365/852 | 42.8 (39.6–46.2) | 775/1,374 | 56.4 (53.8–59.0) | 13.6 (9.3–17.8) |
| Neither registered hypertension nor diabetes | 16/486 | 3.3 (2.0–5.3) | 11/584 | 1.9 (1.1–3.3) | −1.4 (−3.6 to 0.5) |
| Registered hypertension and/or diabetes | 349/366 | 95.4 (92.7–97.1) | 764/790 | 96.7 (95.2–97.7) | 1.4 (−0.9 to 4.2) |

**Notes:** Field presence was any nonblank text in the routine main-medication field and did not indicate verified medication exposure, medication-list completeness, or clinical accuracy. Hypertension and diabetes categories represented routine chronic-disease management registration and not independently adjudicated diagnoses. Proportions have Wilson 95% confidence intervals; PA+ minus PA− differences are percentage points with Newcombe hybrid-score 95% confidence intervals. Wilson and Newcombe intervals are descriptive and do not account for within-site clustering. No hypothesis tests or multiplicity-adjusted comparisons were performed.

**Abbreviations:** CI, confidence interval; PA, routine-recorded physical activity based on the routine exercise-frequency item. PA+ indicates daily exercise, exercise at least once per week, or occasional exercise; PA− indicates no exercise.

**Supplementary Table S4. Observed main-medication-field presence within mutually exclusive hypertension–diabetes management-registration categories**

**Four-category descriptive results**

| Registration category | PA−: n/N; field presence, % (95% CI) | PA+: n/N; field presence, % (95% CI) | PA+ minus PA−, percentage points (95% CI) |
| --- | --- | --- | --- |
| Neither registered hypertension nor diabetes | 16/486; 3.3 (2.0–5.3) | 11/584; 1.9 (1.1–3.3) | −1.4 (−3.6 to 0.5) |
| Registered hypertension only | 284/297; 95.6 (92.7–97.4) | 561/584; 96.1 (94.2–97.4) | 0.4 (−2.2 to 3.7) |
| Registered diabetes only | 6/10; 60.0 (31.3–83.2) | 25/27; 92.6 (76.6–97.9) | 32.6 (4.4–61.8) |
| Both registered hypertension and diabetes | 59/59; 100.0 (93.9–100.0) | 178/179; 99.4 (96.9–99.9) | −0.6 (−3.1 to 5.6) |

**Notes:** The four categories are mutually exclusive. Field presence indicates nonblank routine text and does not establish verified medication exposure, medication-list completeness, or clinical accuracy. Proportions have Wilson 95% confidence intervals, and differences are percentage points with Newcombe hybrid-score 95% confidence intervals. Wilson and Newcombe intervals are descriptive and do not account for within-site clustering. The registered-diabetes-only category contained only 10 PA− and 27 PA+ records and was interpreted descriptively. No formal interaction model or multiplicity-adjusted comparison was retained.

**Abbreviations:** CI, confidence interval; PA, routine-recorded physical activity based on the routine exercise-frequency item. PA+ indicates daily exercise, exercise at least once per week, or occasional exercise; PA− indicates no exercise.

**Supplementary Table S5. Sensitivity analyses and model diagnostics for the four-category Model 3 specification**

**Panel A. Primary and alternative variance estimators**

| Analysis | Examination records, N | Field-present records, n | PR (95% CI) | P value |
| --- | --- | --- | --- | --- |
| Primary four-category Model 3, CR2/Satterthwaite | 2,224 | 1,139 | 1.002 (0.959–1.046) | 0.932 |
| Model 3, HC0 | 2,224 | 1,139 | 1.002 (0.964–1.041) | 0.935 |
| Model 3, HC3 | 2,224 | 1,139 | 1.002 (0.963–1.042) | 0.935 |
| Model 3, site-cluster CR1 | 2,224 | 1,139 | 1.002 (0.963–1.041) | 0.931 |

**Panel B. Alternative registration, PA, covariate, and record-handling specifications**

| Analysis | Examination records, N | Field-present records, n | PR (95% CI) | P value |
| --- | --- | --- | --- | --- |
| Legacy additive hypertension and diabetes registration coding | 2,224 | 1,139 | 1.014 (0.962–1.069) | 0.547 |
| Prior PA harmonization (weekly exercise category excluded) | 2,213 | 1,132 | 0.998 (0.954–1.045) | 0.940 |
| Daily exercise versus PA− | 2,167 | 1,101 | 0.998 (0.952–1.046) | 0.909 |
| Records with missing PA coded as PA− | 2,268 | 1,140 | 1.004 (0.962–1.048) | 0.830 |
| Weekly or frequent alcohol-use covariate | 2,224 | 1,139 | 1.003 (0.960–1.047) | 0.892 |
| Original BMI values retained | 2,226 | 1,140 | 1.001 (0.960–1.043) | 0.964 |
| Exact duplicate examination records excluded | 2,222 | 1,138 | 1.002 (0.959–1.046) | 0.937 |
| Sites with fewer than 20 records excluded | 2,204 | 1,128 | 1.001 (0.959–1.046) | 0.943 |

**Panel C. Leave-one-original-examination-site-out analyses**

| Excluded original examination site | Examination records, N | Field-present records, n | PR (95% CI) | P value |
| --- | --- | --- | --- | --- |
| Site 01 | 1,832 | 906 | 1.012 (0.960–1.066) | 0.621 |
| Site 02 | 1,920 | 1,012 | 0.993 (0.952–1.036) | 0.719 |
| Site 03 | 1,924 | 1,000 | 1.012 (0.966–1.061) | 0.551 |
| Site 04 | 1,958 | 989 | 1.007 (0.959–1.058) | 0.742 |
| Site 05 | 2,028 | 1,050 | 1.007 (0.959–1.058) | 0.731 |
| Site 06 | 2,032 | 1,035 | 0.999 (0.953–1.047) | 0.959 |
| Site 07 | 2,042 | 1,046 | 0.997 (0.953–1.043) | 0.891 |
| Site 08 | 2,085 | 1,069 | 0.994 (0.953–1.036) | 0.732 |
| Site 09 | 2,093 | 1,069 | 0.998 (0.955–1.042) | 0.898 |
| Site 10 | 2,122 | 1,086 | 1.000 (0.955–1.048) | 0.984 |
| Site 11 | 2,215 | 1,133 | 1.001 (0.959–1.046) | 0.941 |
| Site 12 | 2,222 | 1,138 | 1.002 (0.959–1.046) | 0.932 |
| Site 13 | 2,222 | 1,138 | 1.002 (0.959–1.046) | 0.923 |
| Site 14 | 2,223 | 1,139 | 1.002 (0.959–1.046) | 0.935 |
| Site 15 | 2,223 | 1,138 | 1.002 (0.959–1.046) | 0.932 |
| Site 16 | 2,223 | 1,139 | 1.002 (0.959–1.046) | 0.935 |
| Site 17 | 2,223 | 1,138 | 1.002 (0.959–1.046) | 0.934 |
| Site 18 | 2,223 | 1,139 | 1.002 (0.959–1.046) | 0.935 |
| Site 19 | 2,223 | 1,139 | 1.002 (0.959–1.046) | 0.934 |
| Site 20 | 2,223 | 1,138 | 1.002 (0.959–1.046) | 0.932 |

**Panel D. Modified Poisson convergence and fitted-value diagnostics**

| Model | Examination records, N | Field-present records, n | Converged | Maximum fitted value | Fitted values >1, n | Interpretation |
| --- | --- | --- | --- | --- | --- | --- |
| M0 | 2,226 | 1,140 | Yes | 0.564 | 0 | No fitted values exceeded 1 |
| M1 | 2,226 | 1,140 | Yes | 0.745 | 0 | No fitted values exceeded 1 |
| M2 | 2,226 | 1,140 | Yes | 1.065 | 148 | Diagnostic only; not interpreted as individual probabilities |
| M3 | 2,224 | 1,139 | Yes | 1.080 | 194 | Diagnostic only; marginal probabilities came from logistic standardization |

**Notes:** Except for explicitly stated alternative PA definitions, prevalence ratios compare PA+ with PA− examination records. The Model 3 specification included age, sex, examination-site fixed effects, four-category hypertension–diabetes management-registration background, cleaned BMI, current smoking, and recorded alcohol use. Primary inference used CR2 cluster-robust covariance with Satterthwaite small-sample degrees of freedom across all 20 original examination sites.

Panel A compares the primary CR2 result with HC0, HC3, and site-cluster CR1 covariance estimators. Panel B evaluates the legacy additive registration coding, alternative PA definitions, classification of missing PA as PA−, alternative alcohol and BMI specifications, duplicate-record handling, and exclusion of sites with fewer than 20 records. Under the prior PA harmonization, daily and occasional exercise were PA+, no exercise was PA−, and the 11 records coded as exercise at least once per week were excluded. The alternative registration coding was retained to document robustness after the four-category specification was finalized.

Panel C sequentially excludes each of the 20 original examination sites while retaining the same four-category Model 3 specification and CR2/Satterthwaite inference. Original sites were not pooled for cluster-robust or influence analyses. Site labels are anonymized and must not be interpreted as rankings of documentation quality or service performance.

All modified Poisson models converged. Fitted values above one were treated solely as diagnostics and not as individual probabilities. The logistic Model 3 used for marginal standardization converged without non-finite coefficients, boundary estimates, or numerical separation warnings; all model-based predicted probabilities were bounded between zero and one. Marginal probabilities and contrasts were estimated independently using logistic marginal standardization. Sensitivity analyses were descriptive and were not treated as separate confirmatory tests. The complementary post hoc scenario coding all missing PA values as PA+ is reported in Supplementary Table S10.

**Abbreviations:** BMI, body mass index; CI, confidence interval; CR1 and CR2, cluster-robust covariance estimators; HC0 and HC3, heteroskedasticity-consistent covariance estimators; M0–M3, sequential models; PA, routine-recorded physical activity based on the routine exercise-frequency item; PR, prevalence ratio. PA+ indicates daily exercise, exercise at least once per week, or occasional exercise; PA− indicates no exercise.

**Supplementary Table S6. Version-controlled structural audit of nonblank routine main-medication-field entries**

**Panel A. Record- and entry-level audit flow**

| **Audit item** | **Count or n/N (%)** | **Interpretation** |
| --- | --- | --- |
| Examination records with a nonblank main-medication field | 1,141/2,270 (50.3%) | Record-level field-presence indicator |
| Raw parsed medication entries | 1,388 | Before removal of exact duplicate complete entry strings within records |
| Exact duplicate complete entry strings removed | 20/1,388 (1.4%) | Programmatic within-record deduplication only |
| Retained medication entries | 1,368 | Entry-level denominator for the structural audit |
| Field-present records contributing at least one retained entry | 1,141/1,141 (100.0%) | All nonblank fields yielded at least one retained entry |
| Records with exactly 1 retained entry | 962/1,141 (84.3%) | Descriptive entry-count structure |
| Records with exactly 2 retained entries | 145/1,141 (12.7%) | Descriptive entry-count structure |
| Records with exactly 3 retained entries | 22/1,141 (1.9%) | Descriptive entry-count structure |
| Records with exactly 4 retained entries | 10/1,141 (0.9%) | Descriptive entry-count structure |
| Records with exactly 5 retained entries | 2/1,141 (0.2%) | Descriptive entry-count structure |

**Panel B. Programmatic structural indicators among 1,368 retained medication entries**

| **Programmatic indicator** | **n/N (%)** | **Permitted interpretation** |
| --- | --- | --- |
| Technical dictionary assignment generated | 1,368/1,368 (100.0%) | Coverage of observed strings by the current rule-based dictionary; not validated medication-name accuracy |
| Provisional medication-name-assignment flag | 101/1,368 (7.4%) | Programmatic uncertainty retained for human review |
| Quantity–unit token identified | 1,357/1,368 (99.2%) | Text-syntax feature; not evidence of a correct clinical dose |
| Final technical dose-syntax screen passed | 1,323/1,368 (96.7%) | Conformance with parser version 2; not clinical dose validation |
| Zero or negative numeric dose flag | 1/1,368 (0.1%) | Programmatic anomaly flag |
| Large solid-unit dose flag | 33/1,368 (2.4%) | Programmatic anomaly flag based on the final rule |
| Duration containing an explicit time unit | 537/1,368 (39.3%) | Structurally interpretable duration text |
| Bare number without an explicit time unit | 615/1,368 (45.0%) | Semantically ambiguous duration text |
| Administration timing or frequency in duration field | 212/1,368 (15.5%) | Text belonging to a different medication-information domain |
| Other or uninterpretable duration text | 4/1,368 (0.3%) | Unresolved text retained for review |
| Synthetic parser test suite passed | 48/48 (100.0%) | Technical test coverage; not clinical validation |
| Retained-entry technical QC reproduced | 1,368/1,368 (100.0%) | Deterministic row-level reproducibility of implemented rules |
| Development-stage joint pharmacist consultation | Yajuan Wang and Dehui Zhang (2 pharmacists) | Joint development-stage review without reviewer-separated decisions; the separate revision-specific assessment is reported in Table S12 |

**Notes:** The unit in Panel A alternates between examination records and parsed medication entries as stated. Only exact duplicate complete medication-entry strings within the same examination record were removed. No examination records were removed during the medication-text audit.

The current dictionary was constructed to cover strings observed in this dataset. Complete technical assignment represents dictionary coverage and not independently validated medication-name normalization. Quantity–unit and technical dose-syntax indicators describe deterministic text conformance and do not establish dose correctness, plausibility, appropriateness, or prescription concordance.

The four observed duration-text categories were mutually exclusive and included all 1,368 retained entries. A bare number without a time unit was structurally ambiguous because its temporal meaning could not be established from text alone.

The number of retained entries within a field-present record must not be interpreted as the number of medications currently used. One retained entry does not establish monotherapy, and multiple retained entries do not establish polypharmacy. The source field was intended to record principal medications during the preceding year among people receiving long-term treatment for chronic disease, not necessarily a complete current-medication list.

The original development-stage consultation by pharmacists Yajuan Wang and Dehui Zhang was joint and did not retain reviewer-separated decisions. The separate revision-specific manual assessment is reported in Supplementary Methods S1.14 and Table S12. No independent prescription, dispensing, medication-container, or structured medication-reconciliation source was available. Technical reproducibility and recorded-text concordance therefore do not establish actual medication exposure, medication-list completeness, clinical dose validity, adherence, persistence, appropriateness, effectiveness, or safety.

**Abbreviations:** None.

**Supplementary Table S7. Comparison of previous publications derived from the same or substantially overlapping 2025 Wangkui County health-examination platform**

| Publication | Sample | Primary question/outcome | Role of medication information | Distinction from present study |
| --- | --- | --- | --- | --- |
| Zhao et al. (2026b) | N = 2,270 descriptive; N = 2,194 model | Labour-type PA and alcohol-use frequency in relation to measured hypertension; secondary lipid analyses | Antihypertensive use informed a supplementary exclusion | Disease-outcome study; no source-field audit |
| Gao et al. (2026) | N = 2,191 | PA index and obesity–metabolic phenotypes in relation to hypertension, electrocardiographic abnormalities, and prediction | Not a primary exposure or outcome | Metabolic-phenotype study; no field audit |
| Zhang et al.  (2026) | N = 2,258 | Labour-type PA and diabetes burden in relation to triglyceride–glucose and urinary-screening outcomes | Glucose-lowering indicators informed definitions | Diabetes-screening study; no field-validity analysis |
| Guo et al. (2026) | Source N = 2,270; classified N = 2,215 | Routine-recorded PA and screening-defined cardio-renal-metabolic vulnerability | Treatment indicators informed screening domains | Screening study; no documentation-context audit |
| Liu et al. (2026) | N = 1,163 women | Diabetes-related exposure and contextual PA in relation to screening-derived abnormality burden | Glucose-lowering use and polypharmacy informed variables | Women-only study; no provenance or text audit |
| Present study | Submission; source N = 2,270; PA N = 2,226; Model 3 N = 2,224; 1,141 nonblank records; 1,368 entries | Medication-field fitness and text structure, with PA used as a recorded behavioral comparison | Medication information was the primary research object | Data-usability study using an overlapping source platform |

**Notes:** All six analyses used the same or a substantially overlapping 2025 Wangkui County annual health-examination source platform. Differences in analytic sample size reflect study-specific eligibility criteria, variable availability, complete-record selection, subgroup restriction, and data-cleaning rules.

The present study is not based on an independent cohort. Its distinct contribution is the direct evaluation of the routine main-medication field as a data object, including record-level field presence and entry-level medication-text structure. Routine-recorded physical activity was used as a recorded behavioral comparison in evaluating secondary field reuse.

Medication-related indicators used in previous publications should not be assumed to be equivalent across studies. The present analysis neither retrospectively validates nor invalidates medication-derived exposure, exclusion, or composite-outcome variables used in the earlier publications. It evaluates whether the underlying routine main-medication field can support broader secondary use without additional provenance assessment and external validation.

An ethics-information correction to the Zhao et al. hypertension report was published as DOI 10.3389/fpubh.2026.1821356; its scientific analyses and original article DOI were unchanged. PA denotes physical activity.

Supplementary Table S8. Exploratory management-registration and medication-class correspondence using the archived dictionary

This post hoc target-class analysis uses archived mappings; the separate revision-specific manual assessment is reported in Table S12. Provisional or unresolved mappings were treated conservatively as unknown. A corresponding class means a class identified in the recorded text, not verified use or appropriate treatment. Hypertension and diabetes rows overlap because dual-registered records contribute to both. The target classes are the confirmed archived antihypertensive label for hypertension and glucose-lowering label for diabetes; they are project-specific classifications (Supplementary Methods S1.13).

Panel A. All registered records

| **Registration** | **Recorded classification** | **n/N** | **%** |
| --- | --- | --- | --- |
| HTN | Corresponding target class identified | 937/1120 | 83.7% |
| HTN | No corresponding target class identified in resolved recorded text | 69/1120 | 6.2% |
| HTN | Indeterminate | 77/1120 | 6.9% |
| HTN | Field blank | 37/1120 | 3.3% |
| DM | Corresponding target class identified | 167/276 | 60.5% |
| DM | No corresponding target class identified in resolved recorded text | 91/276 | 33.0% |
| DM | Indeterminate | 11/276 | 4.0% |
| DM | Field blank | 7/276 | 2.5% |
| Any HTN/DM | At least one target class corresponding to at least one registered condition identified | 1031/1157 | 89.1% |
| Any HTN/DM | No corresponding target class identified in resolved recorded text | 14/1157 | 1.2% |
| Any HTN/DM | Indeterminate | 69/1157 | 6.0% |
| Any HTN/DM | Field blank | 43/1157 | 3.7% |

Panel B. Records with nonblank fields

| **Registration** | **Recorded classification** | **n/N** | **%** |
| --- | --- | --- | --- |
| HTN | Corresponding target class identified | 937/1083 | 86.5% |
| HTN | No corresponding target class identified in resolved recorded text | 69/1083 | 6.4% |
| HTN | Indeterminate | 77/1083 | 7.1% |
| DM | Corresponding target class identified | 167/269 | 62.1% |
| DM | No corresponding target class identified in resolved recorded text | 91/269 | 33.8% |
| DM | Indeterminate | 11/269 | 4.1% |
| Any HTN/DM | At least one target class corresponding to at least one registered condition identified | 1031/1114 | 92.5% |
| Any HTN/DM | No corresponding target class identified in resolved recorded text | 14/1114 | 1.3% |
| Any HTN/DM | Indeterminate | 69/1114 | 6.2% |

Panel C. Both hypertension and diabetes registration

| **HTN target-class status** | **DM target-class status** | **n/N** | **%** |
| --- | --- | --- | --- |
| Identified | Identified | 73/239 | 30.5% |
| Identified | Not identified in resolved recorded text | 89/239 | 37.2% |
| Identified | Indeterminate | 6/239 | 2.5% |
| Not identified in resolved recorded text | Identified | 56/239 | 23.4% |
| Not identified in resolved recorded text | Not identified in resolved recorded text | 1/239 | 0.4% |
| Indeterminate | Identified | 8/239 | 3.3% |
| Indeterminate | Indeterminate | 5/239 | 2.1% |
| Field blank | Field blank | 1/239 | 0.4% |
| Total | Mutually exclusive joint states | 239/239 | 100.0% |

Marginal summaries (overlapping; not additional rows in the total): hypertension target class identified, 168/239 (70.3%); diabetes target class identified, 137/239 (57.3%); both target classes identified, 73/239 (30.5%). The margins reconcile as 769 + 168 = 937 for hypertension and 30 + 137 = 167 for diabetes. Joint combinations not displayed have zero records; the complete state grid is archived.

Notes: Confirmed target-class identification was retained despite another unresolved string. Without a confirmed target label, any provisional or unresolved mapping made that target indeterminate. No corresponding target class identified in resolved recorded text does not establish nonuse. Blank fields are separate. For any registration, identification requires at least one class corresponding to at least one registered condition, not identification for every registered condition. See Supplementary Methods S1.13.

Supplementary Table S9. Additional audit of registered records with blank medication fields

Panel A. Registration subtype

| **Registration** | **Blank / registered N** | **Blank %** | **Wilson 95% CI (%)** |
| --- | --- | --- | --- |
| HTN only | 36/881 | 4.1 | 3.0–5.6 |
| DM only | 6/37 | 16.2 | 7.7–31.1 |
| Both | 1/239 | 0.4 | 0.1–2.3 |
| Any registration | 43/1157 | 3.7 | 2.8–5.0 |

Panel B. All 20 original examination sites

| **Original site** | **Blank / registered N** | **Blank %** | **Wilson 95% CI (%)** |
| --- | --- | --- | --- |
| Site 01 | 9/238 | 3.8 | 2.0–7.0 |
| Site 02 | 9/134 | 6.7 | 3.6–12.3 |
| Site 03 | 7/141 | 5.0 | 2.4–9.9 |
| Site 04 | 3/150 | 2.0 | 0.7–5.7 |
| Site 05 | 3/89 | 3.4 | 1.2–9.4 |
| Site 06 | 3/106 | 2.8 | 1.0–8.0 |
| Site 07 | 3/96 | 3.1 | 1.1–8.8 |
| Site 08 | 4/69 | 5.8 | 2.3–14.0 |
| Site 09 | 0/70 | 0.0 | 0.0–5.2 |
| Site 10 | 2/53 | 3.8 | 1.0–12.8 |
| Site 11 | 0/6 | 0.0 | 0.0–39.0 |
| Site 12 | 0/1 | 0.0 | 0.0–79.3 |
| Site 13 | 0/1 | 0.0 | 0.0–79.3 |
| Site 14 | 0/0 | Not applicable | Not applicable |
| Site 15 | 0/1 | 0.0 | 0.0–79.3 |
| Site 16 | 0/0 | Not applicable | Not applicable |
| Site 17 | 0/1 | 0.0 | 0.0–79.3 |
| Site 18 | 0/0 | Not applicable | Not applicable |
| Site 19 | 0/0 | Not applicable | Not applicable |
| Site 20 | 0/1 | 0.0 | 0.0–79.3 |

Panel C. Characteristics of registered records

| **Characteristic** | **Blank field (N = 43)** | **Nonblank field (N = 1,114)** | **Absolute SMD** |
| --- | --- | --- | --- |
| Age, years | 69.9 (4.9) | 72.7 (5.7) | 0.515 |
| Female | 24/43 (55.8%) | 580/1114 (52.1%) | 0.075 |
| PA+ (available PA) | 26/43 (60.5%) | 764/1113 (68.6%) | 0.172 |
| BMI, kg/m² | 25.5 (3.4) | 25.0 (3.3) | 0.153 |
| Current smoking | 7/43 (16.3%) | 135/1114 (12.1%) | 0.119 |
| Any alcohol | 11/43 (25.6%) | 97/1114 (8.7%) | 0.459 |
| HTN only | 36/43 (83.7%) | 845/1114 (75.9%) | 0.197 |
| DM only | 6/43 (14.0%) | 31/1114 (2.8%) | 0.412 |
| Both | 1/43 (2.3%) | 238/1114 (21.4%) | 0.617 |

Notes: This was an additional post hoc descriptive audit. Values are n/N (%) or mean (SD); available PA denominators exclude missing PA, and BMI follows the original plausibility rule. Site labels follow the archived analysis mapping. A zero registered denominator is not assigned a percentage. Wilson intervals assume a binomial proportion and do not account for within-site correlation. No site-level quality ranking or hypothesis testing was undertaken. PA denotes routine-recorded physical activity based on the routine exercise-frequency item; PA+ indicates daily exercise, exercise at least once per week, or occasional exercise, and PA− indicates no exercise.

Supplementary Table S10. Common-sample consistency checks and deterministic PA coding scenarios

Panel A. Modified Poisson estimates in the same 2,224 complete records

| **Stage** | **N** | **PR (95% CI)** | **Interpretation** |
| --- | --- | --- | --- |
| M0 | 2224 | 1.317 (1.076–1.611) | Supplementary common-sample check |
| M1 | 2224 | 1.346 (1.118–1.619) | Supplementary common-sample check |
| M2 | 2224 | 1.001 (0.960–1.043) | Supplementary common-sample check |
| M3 | 2224 | 1.002 (0.959–1.046) | Supplementary common-sample check |

Panel B. Logistic marginal standardization in the same 2,224 complete records

| **Stage** | **Estimand** | **Estimate** | **95% CI** |
| --- | --- | --- | --- |
| M0 | PA− probability | 42.84 | 33.55–52.66 |
| M0 | PA+ probability | 56.41 | 52.01–60.72 |
| M0 | Difference PA+ minus PA− | 13.57 | 4.92–22.23 |
| M0 | Marginal PR | 1.317 | 1.075–1.613 |
| M1 | PA− probability | 42.22 | 37.42–47.18 |
| M1 | PA+ probability | 56.77 | 53.71–59.77 |
| M1 | Difference PA+ minus PA− | 14.54 | 6.61–22.48 |
| M1 | Marginal PR | 1.344 | 1.135–1.593 |
| M2 | PA− probability | 51.22 | 49.78–52.66 |
| M2 | PA+ probability | 51.21 | 50.27–52.15 |
| M2 | Difference PA+ minus PA− | -0.01 | -2.07–2.05 |
| M2 | Marginal PR | 1.000 | 0.960–1.041 |
| M3 | PA− probability | 51.14 | 49.77–52.51 |
| M3 | PA+ probability | 51.26 | 50.15–52.37 |
| M3 | Difference PA+ minus PA− | 0.12 | -2.07–2.30 |
| M3 | Marginal PR | 1.002 | 0.960–1.046 |

Probabilities are percentages and differences are percentage points. All stages use the same 2,224 records. CR2 delta-method intervals use estimand-specific small-sample degrees of freedom. Panel A Poisson PRs and Panel B Logistic marginal PRs are distinct estimates; neither replaces primary Table 2.

Panel C. Deterministic coding scenarios for all 44 missing PA values

| **Scenario** | **N after BMI exclusions** | **PR (95% CI)** | **Interpretation** |
| --- | --- | --- | --- |
| All missing PA coded PA− | 2268 | 1.004 (0.962–1.048) | Coding scenario only |
| All missing PA coded PA+ | 2268 | 0.999 (0.957–1.043) | Coding scenario only |

Both scenarios were selected before inspecting results and are reported. They do not correct missingness or provide strict effect bounds.

Panel D. Observed four-category PA differences

| **Registration** | **PA− nonblank n/N (%)** | **PA+ nonblank n/N (%)** | **Difference (percentage points)** |
| --- | --- | --- | --- |
| Neither registered hypertension nor diabetes | 16/486 (3.3%) | 11/584 (1.9%) | -1.41 |
| Registered hypertension only | 284/297 (95.6%) | 561/584 (96.1%) | 0.44 |
| Registered diabetes only | 6/10 (60.0%) | 25/27 (92.6%) | 32.59 |
| Both registered hypertension and diabetes | 59/59 (100.0%) | 178/179 (99.4%) | -0.56 |

Differences use PA+ minus PA−; proportion intervals are in Table S4. Diabetes-only records are sparse (10 PA−, 27 PA+). No new confirmatory interaction test was specified. PA denotes routine-recorded physical activity based on the routine exercise-frequency item; PA+ indicates daily exercise, exercise at least once per week, or occasional exercise, and PA− indicates no exercise.

# Supplementary Methods S1.14 Revision-specific manual concordance assessment

A simple random sample of 200 of 1,368 retained medication entries was selected before manual review. Two pharmacists independently reviewed the original text while masked to parser outputs and each other’s initial decisions.

Review domains distinguished direct text extraction from semantic normalization and included drug-name extraction, standardized medication naming, name and ingredient-normalization certainty, broad therapeutic class, route, frequency, dose representation and unit, quantity-unit interpretation, duration category, and adherence.

Initial reviewer-specific decisions were retained before inter-reviewer agreement was calculated. Disagreements were resolved while parser output remained masked. The adjudicated manual reference was then locked before comparison with the frozen parser.

The assessment evaluated text-level concordance only. No independent prescription, dispensing, medication-container, or medication-reconciliation source was available. One dose-range edge case identified in the targeted audit was retained as a documented parser limitation rather than used to tune the parser for the present analysis.

# Supplementary Table S11. Broad therapeutic classes represented in nonblank routine main-medication fields

Panel A. Broad therapeutic classes in the frozen archived project dictionary. Provisional/unresolved mappings are classified as indeterminate, even when an archived provisional class label exists.

| **Archived broad class** | **Retained entries n/N (%)** | **Nonblank records containing class n/N (%)** |
| --- | --- | --- |
| Antihypertensive | 1,038/1,368 (75.9%) | 954/1,141 (83.6%) |
| Glucose-lowering | 199/1,368 (14.5%) | 173/1,141 (15.2%) |
| Other cardiovascular | 7/1,368 (0.5%) | 7/1,141 (0.6%) |
| Other medications | 23/1,368 (1.7%) | 22/1,141 (1.9%) |
| Indeterminate | 101/1,368 (7.4%) | 96/1,141 (8.4%) |

Entry classes are mutually exclusive and sum to 1,368. A record can contain multiple classes, including both a confirmed class and an unresolved entry, so record-level percentages need not sum to 100%. Counts describe recorded text; they are not medication-use prevalence or medication counts. No new ingredient decomposition or ATC assignment was performed.

Panel A continued. Archived standardized names within the two other-medication groups.

| **Archived broad class** | **Archived standardized name** | **Entries n** | **Records n** |
| --- | --- | --- | --- |
| Other cardiovascular | 辛伐他汀 | 2 | 2 |
| Other cardiovascular | 阿司匹林 | 5 | 5 |
| Other medications | 中药 | 1 | 1 |
| Other medications | 丹参片 | 1 | 1 |
| Other medications | 利培酮 | 2 | 2 |
| Other medications | 双环醇 | 7 | 7 |
| Other medications | 异烟肼 | 1 | 1 |
| Other medications | 护肝片 | 1 | 1 |
| Other medications | 替米 | 4 | 4 |
| Other medications | 氯氮平 | 1 | 1 |
| Other medications | 消糖复胰丸 | 1 | 1 |
| Other medications | 肺宁颗粒 | 1 | 1 |
| Other medications | 苯妥英钠 | 1 | 1 |
| Other medications | 软肝胶囊 | 1 | 1 |
| Other medications | 银杏叶片 | 1 | 1 |

Archived mapping certainty in the frozen dictionary.

| **Archived mapping status** | **Retained entries n/N (%)** | **Nonblank records n/N (%)** |
| --- | --- | --- |
| Existing mapping confirmed | 1,267/1,368 (92.6%) | 1,071/1,141 (93.9%) |
| Confirmed provisional/unresolved status | 101/1,368 (7.4%) | 96/1,141 (8.4%) |

Records may contain both confirmed and provisional/unresolved entries, so record-level categories overlap and their percentages need not sum to 100%. These are the two archived parser/dictionary certainty states, separate from the recorded-name certainty and ingredient-normalization certainty assessed in the manual review.

# Supplementary Table S12. Revision-specific manual concordance assessment

Panel A. Manual-validation sample

| **Item** | **Result** |
| --- | --- |
| Retained medication entries | 1,368 |
| Randomly reviewed entries | 200 |
| Sampling fraction | 14.6% |
| Reviewers | 2 pharmacists |
| Initial review masked to parser | Yes |
| Reviewer-specific decisions retained | Yes |
| Disagreements resolved before parser unblinding | Yes |

Panel B. Inter-reviewer agreement

| **Domain** | **n/N** | **Agreement %** | **95% CI** | **κ** |
| --- | --- | --- | --- | --- |
| Drug-name extraction | 200/200 | 100.0 | 98.1–100.0 | — |
| Standardized name | 181/200 | 90.5 | 85.6–93.8 | — |
| Ingredient-normalization certainty | 192/200 | 96.0 | 92.3–98.0 | 0.418 |
| Broad therapeutic class | 199/200 | 99.5 | 97.2–99.9 | 0.982 |
| Route interpretability | 197/200 | 98.5 | 95.7–99.5 | — |
| Quantity-unit interpretation | 197/200 | 98.5 | 95.7–99.5 | — |
| Duration category | 200/200 | 100.0 | 98.1–100.0 | 1.000 |

Notes: Agreement confidence intervals are Wilson intervals. Recorded-name certainty, route raw-token extraction, frequency, dose representation, dose value/bounds, dose unit, and adherence text each had 200/200 agreement (100.0%; 95% CI 98.1–100.0). Agreement on absent values does not establish successful extraction. Unweighted kappa 95% percentile intervals from 5,000 paired-entry bootstrap resamples (seed 20260917) were 0.000–0.707 for ingredient-normalization certainty, 0.943–1.000 for broad therapeutic class, and 1.000–1.000 for duration category; all 5,000 resamples were defined for each domain. Ingredient-normalization certainty was Confirmed/Provisional/Unresolved in 197/2/1 entries for reviewer A and 189/7/4 for reviewer B. Indeterminate judgments and all confidence levels were retained. A dash indicates that kappa was not reported for that domain.

Panel C. Parser versus locked pharmacist reference

| **Domain** | **Concordant n/N** | **Concordance %** | **95% CI** |
| --- | --- | --- | --- |
| Drug-name extraction | 200/200 | 100.0 | 98.1–100.0 |
| Standardized medication name | 155/200 | 77.5 | 71.2–82.7 |
| Broad therapeutic class | 198/200 | 99.0 | 96.4–99.7 |
| Quantity-unit interpretation | 200/200 | 100.0 | 98.1–100.0 |
| Duration category | 200/200 | 100.0 | 98.1–100.0 |

Notes: The locked pharmacist reference was finalized before parser output was unblinded. Concordance represents exact agreement between the frozen parser and the adjudicated manual reference after Unicode NFKC normalization and trimming of outer whitespace. Missing and indeterminate states were retained as valid categories. These measures assess recorded-text concordance and do not represent sensitivity, specificity, predictive value, or clinical medication-exposure accuracy.

Drug-name extraction used the saved raw drug-name field. Standardized-name comparison used the saved dictionary label and the adjudicated manual name without new synonym substitution, salt removal, dosage-form removal, or ingredient remapping. Broad-class comparison used the saved therapeutic-group label with fixed language-label equivalents; mapping certainty was assessed separately. This comparison differs from the conservative S11 descriptive grouping, which classifies provisional/unresolved mappings as indeterminate. Quantity-unit comparison concerned Present, Absent, or Indeterminate status, not equality of dose values or scalar/range shape; a recorded numeric range with a recognized unit remained Present. Duration labels were reconciled only by fixed equivalents for the five categories.

Informative-text concordance remained 197/197 (100.0%; Wilson 95% CI 98.1–100.0) among entries whose manual reference classified the quantity-unit structure as Present. Duration concordance among the 198 entries classified as explicit time unit, bare number without unit, or timing/frequency was 198/198 (100.0%; 95% CI 98.1–100.0); the two other/uninterpretable entries were retained in the overall 200-entry estimate. All 200 entries had drug-name text and none had Missing duration. The 45 standardized-name discrepancies and two broad-class discrepancies were retained without changing the reference or frozen parser. The full paired comparison and reference-derivation audit were archived.

# Supplementary Table S13. Common-sample quantitative illustration of naïve field reuse

| **Model** | **PA− apparent %** | **PA+ apparent %** | **Difference pp (95% CI)** | **Marginal PR (95% CI)** |
| --- | --- | --- | --- | --- |
| M0 | 42.84 | 56.41 | 13.57 (4.92 to 22.23) | 1.317 (1.075–1.613) |
| M2 | 51.22 | 51.21 | -0.01 (-2.07 to 2.05) | 1.000 (0.960–1.041) |
| M3 | 51.14 | 51.26 | 0.12 (-2.07 to 2.30) | 1.002 (0.960–1.046) |

All rows use the same 2,224 complete examination records. M0 is unadjusted; M2 includes age, sex, site and the four-category registration context; M3 additionally includes cleaned BMI, smoking and alcohol recording. These are logistic marginal-standardization estimates with the original CR2/Satterthwaite delta-method inference. The illustrative interpretation of field presence as a medication-exposure proxy is deliberately naïve. The table does not establish verified medication prevalence, a causal PA effect, or the amount of exposure misclassification corrected by adjustment.
