# Supplementary material for: Among rural older adults, an apparent physical-activity contrast in nonblank medication-field presence was markedly attenuated when chronic-disease management registration was incorporated into the analysis
Source: Front Pharmacol. 2026 Oct 15;17:1965382. doi: 10.3389/fphar.2026.1965382 (PMC13716648; doi:10.3389/fphar.2026.1965382)
Supplement: Supplementary file 2 [file Supplementaryfile2.docx]

**Supplementary Figure S1. Distribution of routine-recorded exercise-frequency categories and reported exercise minutes per session.** Panel A shows original exercise-frequency categories among all 2,270 populated examination records. Daily exercise, exercise at least once per week, and occasional exercise were classified as PA+; no exercise was PA−; blank values were missing. Percentages use all populated examination records as the denominator. Panel B shows reported exercise minutes per session among 1,366 PA+ records with positive duration. The three most frequent values were 30 minutes (n = 222), 60 minutes (n = 801), and 120 minutes (n = 196). The source field represented intentional exercise and was not a comprehensive measure of total physical activity. PA denotes routine-recorded physical activity based on the routine exercise-frequency item; PA+ indicates daily exercise, exercise at least once per week, or occasional exercise, and PA− indicates no exercise. Concentration at round durations may reflect rounding, reporting, or entry practices; it is not evidence of validated activity exposure.

**Supplementary Figure S2. Observed main-medication-field presence by routine-recorded physical activity across four mutually exclusive management-registration categories.** Points show observed proportions and horizontal bars Wilson 95% confidence intervals. Gray circles represent PA− records and teal squares PA+ records. Labels report percentages and field-present records/total records. Hypertension and diabetes indicate routine chronic-disease management registration rather than independently adjudicated diagnoses. The registered-diabetes-only category had few records, particularly in the PA− group, and was interpreted descriptively. Field presence indicates nonblank routine text and does not establish verified medication exposure, medication-list completeness, or clinical accuracy. PA denotes routine-recorded physical activity based on the routine exercise-frequency item; PA+ indicates daily exercise, exercise at least once per week, or occasional exercise, and PA− indicates no exercise.

**Supplementary Figure S3. Examination-site composition and main-medication-field presence across anonymized analysis-site categories.** Bubble area is proportional to the number of physical-activity-complete records. Sites contributing fewer than 20 records were combined into a pooled small-site category (PS) for fixed-effect adjustment and display only. Panel A plots the percentage with registered hypertension and/or diabetes against field presence; Panel B plots the percentage PA+ against field presence. These displays are descriptive; no site-level correlation or causal relationship was estimated, and labels do not rank documentation quality or service performance. All 20 original examination sites were retained separately for CR2, CR1, and original-site influence analyses. PA denotes routine-recorded physical activity based on the routine exercise-frequency item; PA+ indicates daily exercise, exercise at least once per week, or occasional exercise, and PA− indicates no exercise.

**Supplementary Figure S4. Sensitivity and original-site influence analyses for the Model 3 prevalence-ratio estimate.** Points show prevalence ratios comparing PA+ with PA− records, horizontal lines show 95% confidence intervals, and the dashed line denotes 1.00. Panel A shows the primary four-category Model 3 using CR2 cluster-robust covariance with Satterthwaite small-sample degrees of freedom, the legacy additive registration specification, alternative physical-activity and covariate definitions, missing-activity coding, duplicate-record and small-site handling, and HC0, HC3, and CR1 variance estimators. Under the prior PA harmonization, daily and occasional exercise were PA+, no exercise was PA−, and the 11 records coded as exercise at least once per week were excluded. Panel B sequentially excludes each of the 20 original examination sites while retaining the four-category Model 3 and CR2/Satterthwaite inference. All analyses were descriptive robustness checks. BMI denotes body mass index. PA denotes routine-recorded physical activity based on the routine exercise-frequency item; PA+ indicates daily exercise, exercise at least once per week, or occasional exercise, and PA− indicates no exercise.
